# Characterization of Oncology Clinical Trials Using Germline Genetic Data

**DOI:** 10.1001/jamanetworkopen.2022.42370

**Published:** 2022-11-16

**Authors:** Ashwin V. Kammula, Alejandro A. Schäffer, Padma Sheila Rajagopal

**Affiliations:** 1 Cancer Data Science Laboratory, Center for Cancer Research, National Cancer Institute, Bethesda, Maryland; 2 University of Maryland, College Park; 3 Women’s Malignancies Branch, Center for Cancer Research, National Cancer Institute, Bethesda, Maryland

## Abstract

**Importance:**

The recent successes of poly-ADP ribose polymerase (PARP) inhibitors and belzutifan support germline genetic data as an exciting, accessible source for biomarkers in cancer treatment. This study hypothesizes, however, that most oncology clinical trials using germline data largely prioritize *BRCA1/2* as biomarkers and PARP inhibitors as therapy.

**Objective:**

To characterize past and ongoing oncology trials that use germline data.

**Design, Setting, and Participants:**

This retrospective cross-sectional study of oncology trials used the Informa Trialtrove database to evaluate trial attributes. Trials using germline information (including the terms *germline*, *hereditary*, or *inherited* in the title, treatment plan, interventions, end points, objectives, results, or notes) and conducted globally between December 1, 1990, and April 4, 2022 (data freeze date), were included.

**Main Outcomes and Measures:**

Trials by cancer type, phase, participants, sponsor type, end points, outcomes, and locations were described. Associated biomarkers and mechanisms of action for studied therapeutic interventions were counted. How germline data in trial inclusion and exclusion criteria are associated with end points, outcomes, and enrollment were also examined.

**Results:**

A total of 887 of 84 297 (1.1%) oncology clinical trials in the Trialtrove database that use germline data were identified. Most trials were conducted in cancer types where PARP inhibitors are already approved. A total of 74.8% (672) of trials were performed in the phase 2 setting or above. Trials were primarily sponsored by industry (523 trials [59.0%]), academia (382 trials [43.1%]), and the government (274 trials [30.9%]), where trials may have multiple sponsor types. Among 343 trials using germline data with outcomes in Trialtrove, 180 (52.5%) reported meeting primary end points. Although *BRCA1/2* are the most frequent biomarkers seen (*BRCA1*, 224 trials [25.3%]; *BRCA2*, 228 trials [25.7%]), trials also examine pharmacogenomic variants and germline mediators of somatic biomarkers. PARP inhibitors or immunotherapy were tested in 69.9% of trials; PARP inhibition was the most frequently studied mechanism (367 trials [41.4%]). An overwhelming number of trials using germline data were conducted in the US, Canada, and Europe vs other countries, mirroring disparities in cancer genetics data. Germline data in inclusion and exclusion criteria are associated with altered end point, outcomes, and enrollment compared with oncology trials with no germline data use. Examples of inclusion and exclusion criteria regarding germline data that may unintentionally exclude patients were identified.

**Conclusions and Relevance:**

These findings suggest that for germline biomarkers to gain clinical relevance, trials must expand biomarkers, therapies, and populations under study.

## Key Points

**Question:**

What are features of clinical trials in oncology that use germline data?

**Findings:**

In this cross-sectional study of 84 297 oncology clinical trials included in the Informa Trialtrove databse, 1.1% used germline data, with 52.5% of trials with outcomes available meeting primary end points. Poly-ADP ribose polymerase inhibition was the most frequently studied mechanism (41.4%), whereas *BRCA2* (25.7%) and *BRCA1* (25.3%) were the most frequently studied biomarkers; trials were largely conducted in the US, Canada, and Europe.

**Meaning:**

These findings suggest that for germline biomarkers to gain clinical relevance, trials must expand biomarkers, therapies, and populations under study.

## Teaser

This cross-sectional study evaluates the features of oncology clinical trials that use germline genetic data.

## Introduction

The role of germline genetic data, defined here as inherited or hereditary variants, has changed dramatically in oncology management. Classically, germline data determine the risk of developing cancer associated with familial syndromes (eg, Lynch or Li-Fraumeni syndromes).^1^ Somatic genetic data from tumor tissue provide biomarkers for treatment estimation and prognostication.^2^ However, with the first US Food and Drug Administration approval of a poly-ADP ribose polymerase (PARP) inhibitor for germline-variant *BRCA*-associated ovarian cancer in 2014 and the 2021 approval of belzutifan for germline-variant *VHL*-associated kidney cancer, germline genetic data entered the sphere of treatment-associated biomarkers.^3,4^ Sessions discussing germline variants as predictive were held at the 2022 American Association of Cancer Research and American Society of Clinical Oncology conferences.^5,6^

Given the rapid expansion in the clinical utility of germline data in medical oncology, we report on the landscape of past and ongoing oncology trials using germline data. We hypothesized that, given the success of PARP inhibitors, most trials would focus on variations in genes in the DNA damage repair pathway (such as *BRCA1, BRCA2, *and* PALB2*) as therapeutic biomarkers, and accordingly PARP inhibitors as therapy, rather than expanding to other hereditary cancer syndrome genes.

To develop the first landscape of clinical oncology trials that use germline data and test this hypothesis, we used the Informa Trialtrove database , an online repository of trials that includes ClinicalTrials.gov among more than 58 000 sources.^7,8^ Our study describes these trials, the most frequently studied germline genes and associated treatments, and considerations for future studies.

## Methods

### Trialtrove

This study did not undergo institutional review board review. Informed consent was not needed. This is a secondary analysis of published reports with data at the trial level and so not is considered human participants research in accordance with 45 CFR §46. The Strengthening the Reporting of Observational Studies in Epidemiology (STROBE) reporting guideline for cross-sectional studies was used to ensure accurate reporting.

Data in Trialtrove are presented in a more structured format compared with ClinicalTrials.gov, facilitating searches using formal language methods.^9,10^ Each trial in Trialtrove contains more than 75 fields of data. Structured columns make it possible to categorize data from identified trials using automated computational methods. As Trialtrove access requires a paid license,^11,12^ we can only report summary information.

### Search Strategy

In this retrospective cross-sectional study, we retrieved all oncology trials (84 297 trials) included in Trialtrove with a data freeze date of April 4, 2022. We searched 9 columns for these trials using a Python script (see eMethods in the Supplement). We defined oncology trials as using germline information if the terms *germline*, *hereditary*, or *inherited* were present in the searched columns.

### Trial Analysis

To characterize the portfolio of germline clinical trials, we used as input the subset of trials (887 trials) found by the search strategy detailed in eMethods in the Supplement. Pathways included are from the Gene Ontology: Biological Process set.^13^

To determine risk of inadvertent recruitment exclusion associated with inclusion and exclusion criteria, we applied the Python method re.search() to the inclusion criteria and exclusion criteria free-text fields in Trialtrove for the 887 trials. In addition to the prior search terms, we searched *variant*, *mutation*, *allele*, and *pathogenic* in the inclusion and exclusion fields. Inclusion and exclusion criteria were then reviewed manually and duplicates removed for the resulting subset (322 trials).

### Statistical Analysis

Descriptive analyses of the data were conducted. We performed Cochran-Mantel-Haenszel tests stratified by trial phase to compare categories of primary end points and outcomes between trials that used germline data as part of eligibility criteria and oncology clinical trials with no germline data use (nongermline). Trialtrove reports accrual percentage for each trial. We tabulated the frequency of trials with various ranges of accrual percentages (ie, 0% to 10% accrual, 10% to 20% accrual, …, and ≥100%) in each group and applied a Pearson χ^2^ test to compare accrual rates. *P* < .05 was considered significant in 2-sided tests. Tables and figures were generated using R statistical software version 4.1.2 (R Project for Statistical Computing) except for Figure 3, which was generated in Tableau version 2021.4.3.^14^

## Results

### Attributes of Clinical Trials Involving Germline Data

**Figure 1.**
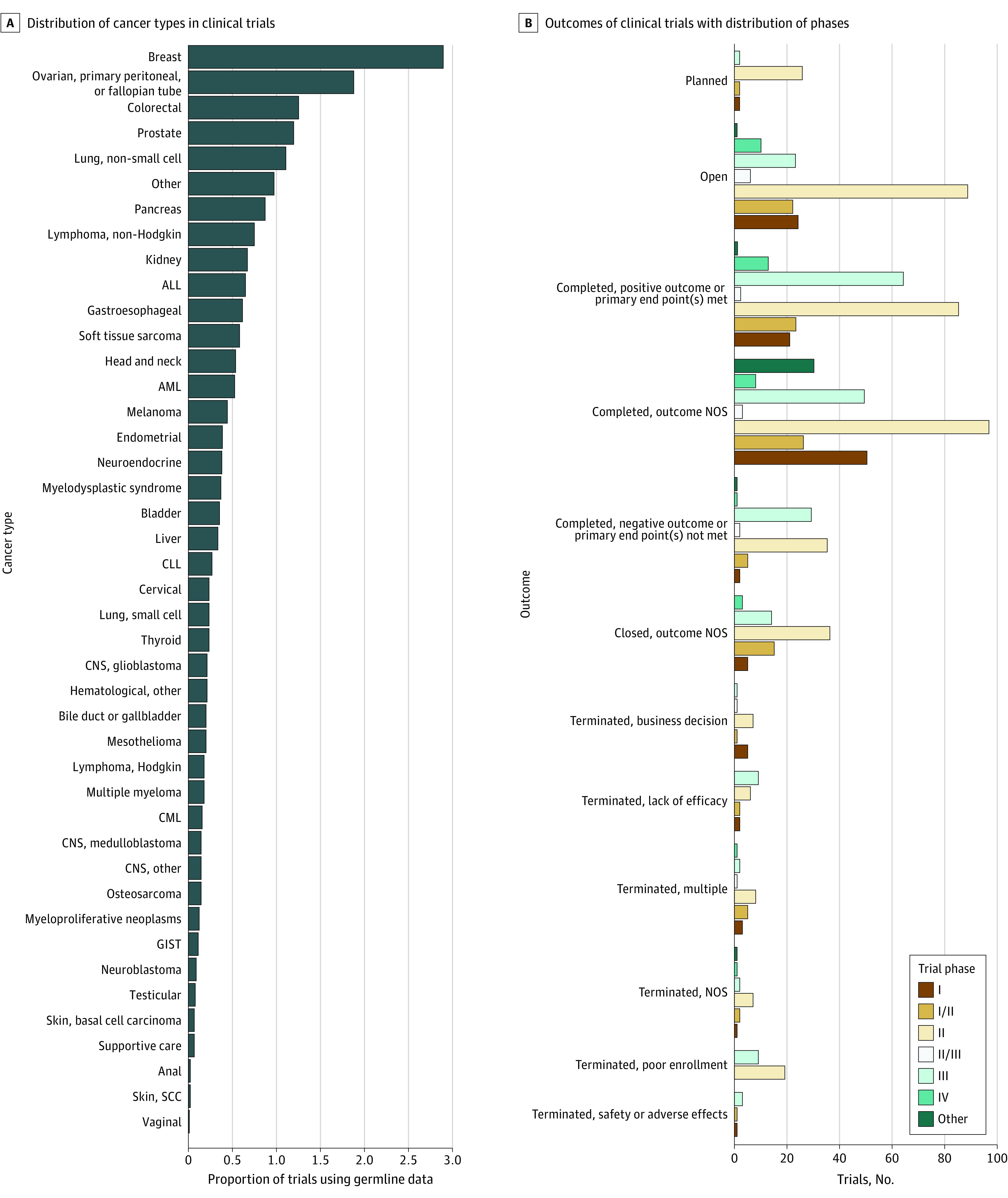
Cancer Types, Designs, and Outcomes of Oncology Clinical Trials Using Germline Data Panel A shows distribution of cancer types associated with clinical trials. Trials may include multiple cancer types. For reporting purposes, cancer types were condensed into single categories as follows: ovarian, primary peritoneal, and fallopian tube cancer into ovarian; unspecified solid tumor, metastatic cancer, N/A, and unspecified cancer into other. Hematological: other included trials with focus on noncancerous diseases such as thalassemia, anemia, and sickle-cell disease, and their association with cancers or treatments as well as unspecified hematologic cancers and transplantation/graft-versus-host disease. See eTable 1 in the Supplement for full counts by cancer type. Panel B shows outcomes of clinical trials with distribution of phases. Each trial can have at most 1 phase and 1 outcome in this classification. Blank, other, unknown, or indeterminate outcomes were listed as not otherwise specified (NOS). Business decisions were aggregated, while these are listed separately in Trialtrove. AML indicates acute myelogenous leukemia; ALL, acute lymphocytic leukemia; CLL, chronic lymphocytic leukemia; CNS, central nervous system; CML, chronic myelogenous leukemia; GIST, gastrointestinal stromal tumor; SCC, squamous cell carcinoma.

Of 84 297 oncology trials included in Trialtrove as of April 4, 2022, 887 (1.1%) included the search terms *germline*, *inherited*, or *hereditary* as part of the fields listed in eMethods in the Supplement (Figure 1 and Table). Although trials occur in at least 42 cancer types (additional, rarer subtypes consolidated under other), most trials include cancer types already FDA-approved for PARP inhibitors, with 259 trials (29.2%) in breast cancer; 168 trials (18.9%) in ovarian, primary peritoneal, and fallopian tube cancer; 107 trials (12.1%) in prostate cancer; and 78 trials (8.8%) in pancreatic cancer. Otherwise, the most frequent cancer types include colorectal (112 trials [12.6%]), non–small cell lung (99 trials [11.2%]), and other solid tumors (87 trials [9.8%]) (Figure 1A and eTable 1 in the Supplement). Percentages add to more than 100 because some trials evaluate multiple cancer types. Most of the 887 trials (651 [73.4%]) include patients with metastatic disease.

**Table.** Descriptive Characteristics of Oncology Clinical Trials That Use Germline Data^a^

Characteristic	Trials, No. (%) (N = 887)
Cancer stages of patients	
Early stage (neoadjuvant or adjuvant)	162 (18.3)
Stage	
I	112 (12.6)
II	178 (20.1)
III	462 (52.1)
IV, advanced, or metastatic	651 (73.4)
Trial phase	
1	114 (12.9)
1/2	101 (11.4)
2	389 (43.7)
2/3	15 (1.7)
3	197 (22.2)
4	35 (4.0)
Other	36 (4.1)
Sponsor type	
Academic	382 (43.1)
Cooperative group	241 (27.2)
Miscellaneous	10 (1.1)
Industry	523 (59.0)
Not for profit	47 (5.3)
Government	274 (30.9)
Trial status	
Planned	16 (1.8)
Open	174 (19.6)
Completed	
Positive outcome or primary end point(s) met	180 (20.3)
Outcome NOS	262 (29.5)
Negative outcome or primary end point(s) not met	75 (8.5)
Closed, outcome NOS	75 (8.5)
Terminated	
Business decisions	15 (1.7)
Lack of efficacy	19 (2.1)
Multiple reasons	20 (2.3)
Reason NOS	14 (1.6)
Poor enrollment	28 (3.2)
Safety or adverse effects	5 (0.6)
Lack of funding	1 (0.1)
No data	3 (0.3)

Abbreviation: NOS, not otherwise specified.

^a^ Each of the 887 trials may include multiple categories of stages or sponsors. However, each trial has exactly 1 phase category, with trials considered to be combination studies denoted as such.

Most trials (672 [75.8%]) were phase 2 or above (Table and Figure 1B). More than half of trials (523 [59.0%]) were sponsored by industry, 382 (43.1%) by academia, and 274 (30.9%) by government (Table). A total of 343 trials (38.7%) have outcome data reported in Trialtrove (eMethods in the Supplement). All but 3 trials had information in either trial outcomes or trial status (Table and Figure 1B); 180 trials (52.5%) were designated as having a positive outcome in Trialtrove (either completed, positive outcome or primary end point[s] met, or completed, early positive outcome) (eMethods in the Supplement). Of these 180 trials, 141 (78.3%) were phase 2 or above (Figure 1B). Reasons that trials were terminated included poor enrollment, lack of efficacy, business decisions, and safety and adverse effects. One trial (not shown in Figure 1B) was terminated due to lack of funding.

### Germline Data Biomarkers Used in Clinical Trials Involving Germline Data 

**Figure 2.**
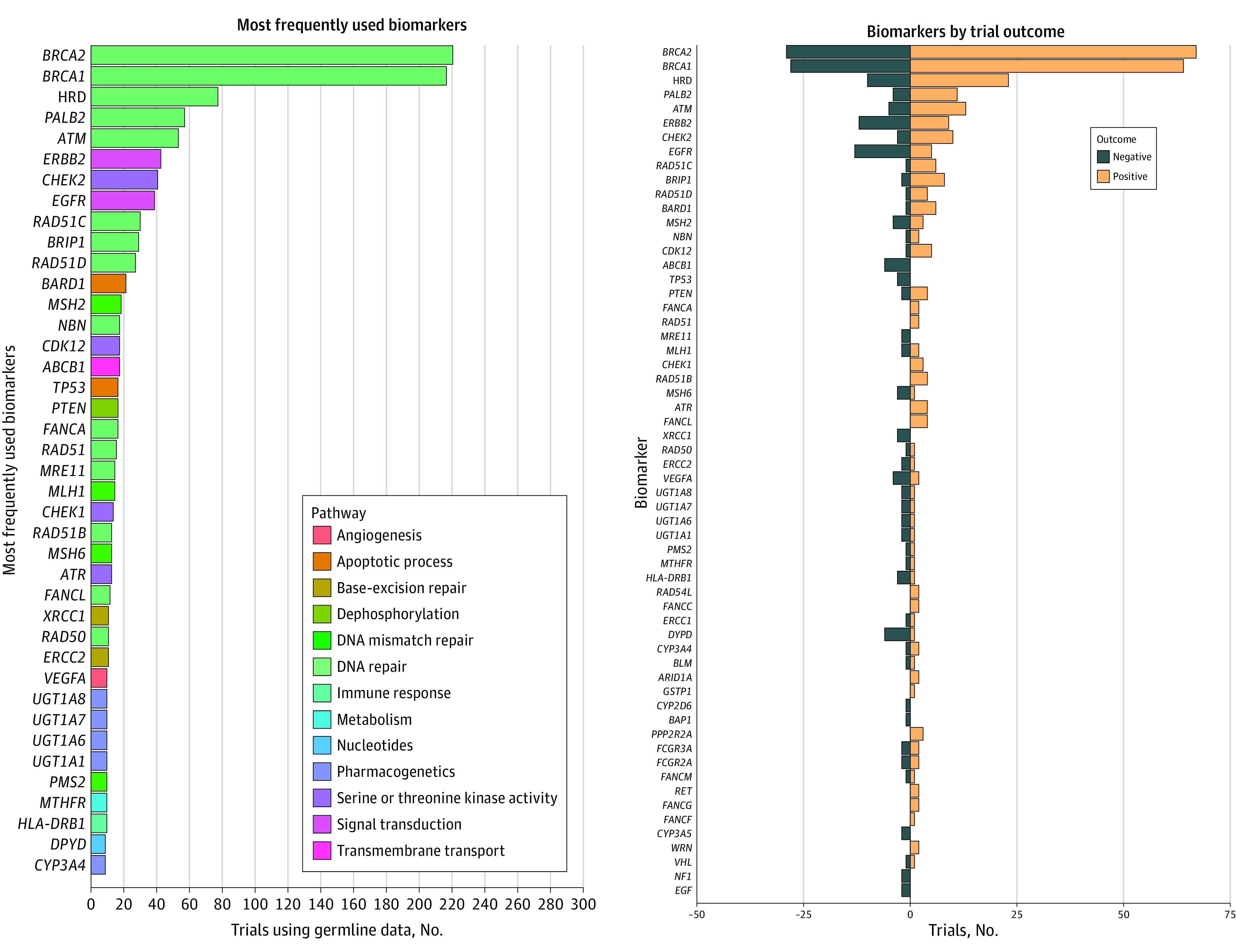
Biomarkers Included in Oncology Clinical Trials Using Germline Data Panel A shows biomarkers most frequently associated with oncology clinical trials using germline data. Most frequent 40 biomarkers shown on figure, with x-axis displaying number of trials in Trialtrove that listed the gene as an oncology biomarker. Rows colored according to associated leading gene ontology biological process pathways, shown in legend on right. Full list of biomarkers in eTable 2 in the Supplement. Panel B shows biomarkers shown by trial outcome where reported. Negative and terminated trials in Trialtrove were aggregated for the purpose of visualization. The x-axis displays number of trials in Trialtrove that listed the gene as an oncology biomarker and a trial outcome that was either positive or negative or terminated. Rows colored by outcome status.

The most common biomarkers studied in clinical trials involving germline data are shown in Figure 2A (eTable 2 in the Supplement). The most frequent genes were *BRCA2* (228 trials [25.7%]) and *BRCA1* (224 trials [25.3%]). Biomarkers were most frequently involved in DNA repair, with the most non-*BRCA* biomarkers being homologous recombination deficiency, measured through any method (80 trials [9.0%]), *PALB2* (59 trials [6.7%]), and *ATM* (55 trials [6.2%]). After DNA repair, biomarkers were most frequently associated with pharmacogenetic pathways.

Among the top 10 biomarkers used in trials, we identified 2 genes typically associated with somatic cancer features. *ERBB2* can be used as a biomarker for ERBB2-targeted treatments and was evaluated in 44 trials (5.0%). *EGFR* can serve as a biomarker for some EGFR-directed therapies and was associated with 40 trials (4.5%). Trials studying *ERBB2* and *EGFR* most often sought germline modifiers of therapeutic response; 5 trials examined germline variants in *EGFR*.

Figure 2B shows the most common biomarkers and positive or negative trial outcomes in Trialtrove. Biomarkers associated with the DNA damage repair pathway demonstrated a higher frequency of positive outcomes than other pathways.

### Therapies Studied in Clinical Trials Using Germline Data

Among the 887 trials in Trialtrove, 885 were listed with a primary therapy or combination of therapies by mechanism of action (eTable 3 in the Supplement). PARP inhibition (367 trials [41.4%]), angiogenesis inhibitors (331 trials [37.3%]), and immunotherapy (253 trials [28.5%]) were the most frequently tested therapies. Overall, PARP inhibitors or immunotherapy were tested in 69.9% of trials. Combinations involving PARP inhibitors were described as the primary intervention in 108 trials, vs 52 trials for angiogenesis inhibitors, and 42 trials for immunotherapy. These findings are consistent with approvals of PARP inhibitor therapy for patients with germline *BRCA1/2* variations. Olaparib, a PARP inhibitor, combined with bevacizumab, an angiogenesis inhibitor via VEGF, is a therapeutic approach in ovarian cancer. Immunotherapy is approved for patients with microsatellite instability potentially arising from germline variations in *MLH1*, *MSH2*, *MSH6*, and *PMS2*.

### Countries Where Clinical Trials Using Germline Data Are Conducted

**Figure 3.**
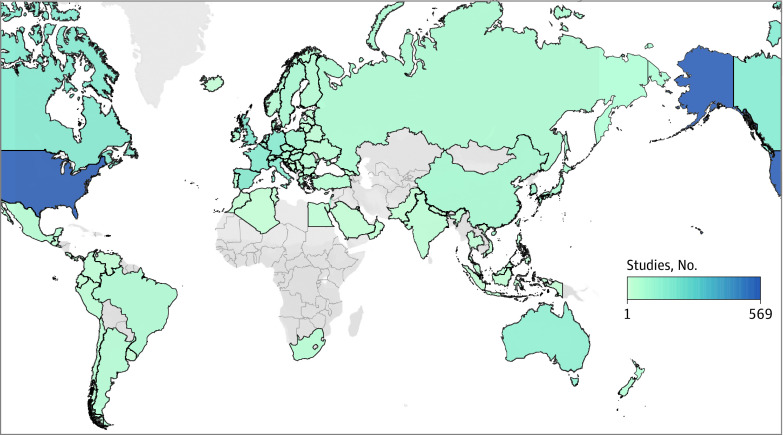
Distribution of Oncology Clinical Trials Using Germline Data by Country Dark blue is indicative of a higher number of studies, while gray is indicative of no studies. Figure generated using Tableau.

Because trials have inconsistent reporting regarding race, and almost no trials collect sufficient genomic data for ancestry, we examined countries in which these trials were being performed, obtaining the distribution visualized in Figure 3. Most studies are being conducted in the US (569 trials [64.2%]). Beyond the US and Canada, the top 10 countries performing clinical trials involving germline data are in Europe. South Korea (90 trials [10.2%]) and China (74 trials [8.3%]) were the best represented Asian countries, Brazil was the best represented South American country (53 trials [6.0%]), and South Africa the best represented African country (30 trials [3.4%]). As trials may be conducted in multiple countries, percentages add to more than 100.

### Association of Germline Inclusion and/or Exclusion Criteria vs No Germline Data Use With Trial Attributes

Our search identified 322 of 887 trials (36.3%) that included germline information in inclusion and/or exclusion criteria. We compared primary end points, recruitment, and outcomes in Trialtrove between these 322 trials and the 83 410 oncology trials that did not use any germline information (nongermline). Trial end points and outcomes showed significant association with eligibility criteria. Germline trials used survival-based end points less frequently than nongermline trials (eFigure 1 in the Supplement). Germline trials also had a higher relative distribution of completed positive studies and open studies (eFigure 2 in the Supplement). Eligibility criteria had a significant association with accrual rate, with a higher proportion of germline trials reporting greater than 100% anticipated accrual compared with nongermline trials (eFigure 3 in the Supplement).

Germline variants from underrepresented populations were more frequently categorized by the 2015 American College of Medical Genetics criteria as variants of uncertain significance, (VUS) in accordance with limited data.^15^ Patients may therefore be excluded if inclusion and exclusion criteria require pathogenic germline variants not directly related to a population being studied or mechanism of action. We sought to determine whether any germline variation requirements may result in inadvertent recruitment exclusions.

Trials were considered at risk for potential recruitment exclusion if (1) inclusion criteria specified ancestry-specific or population-specific variants without focusing on those populations, or (2) if germline variant pathogenicity or likely pathogenicity was required for an interventional study for patients with cancer without additional routes for trial inclusion (investigator review, clinical criteria for syndromes, family history review, and so forth). The latter standard was not applied to studies that sought unaffected carriers of pathogenic or likely pathogenic germline variants for screening or prevention.

Three trials^16,17,18^ explicitly mentioned ancestry-specific inclusion criteria not related to their research questions. Each trial specified that patients of Ashkenazi Jewish ancestry with personal history of breast cancer or family history of cancer were eligible given possible germline *BRCA-*variant status, but none of these trials stipulated that such patients would undergo any risk evaluation or germline testing to confirm variation status before participation. No trials in our search listed specific founder variations as part of inclusion criteria. Of note, 1 trial^19^ stated, “It is expected that *BRCA* testing will be covered as medically necessary by the patient’s insurance carrier.” This specific criterion is associated with disparities in genetic testing.^20,21^ Fifty-three trials (14.7%) required pathogenic germline variations without providing additional routes for patients to be considered eligible for study.

## Discussion

To our knowledge, this cross-sectional study is the first to describe the landscape of oncology trials using germline data. Consistent with our hypothesis, most trials currently using germline data prioritize *BRCA 1/2* and explore additional indications for PARP inhibitors (such as earlier-stage disease) in cancer types where PARP inhibitors already work.

We used Trialtrove for this project. This database not only expands on data available from ClinicalTrials.gov, but also provides annotation and data curation to facilitate computationally driven search strategies. As of July 2022, we found only 1 other oncology trial landscape project that used Trialtrove, a review of neoadjuvant and adjuvant immunotherapy clinical trials.^12^ Although our initial search was similar to that of Wu et al,^12^ our downstream assessment included biomarkers and inclusion and exclusion criteria, as well as treatments under study.

We observe a 52.5% trial success rate among the trials in our study with reported outcomes. In a landmark 2015 study by Wong et al^22^ using Trialtrove data, phase 2 and above oncology trials were estimated to have success rates of 32.7% (95% CI, 31.5%-33.9%) from phase 2 to phase 3 and 35.5% (95% CI, 32.8%-38.2%) from phase 3 to approval. Although the advent of immunotherapy would likely increase estimated success rates from Wong et al,^22^ the researchers reported that study biomarkers improved likelihood of trial success.

Our excitement at this comparison, though, remains tempered by the oncology field’s persistent focus on *BRCA1/2* and PARP inhibitors, which likely exacerbates selection bias. We observe reduced use of survival end points among trials that use germline eligibility criteria compared with nongermline trials and, accordingly, higher relative frequencies of trials reaching positive outcomes compared with the broader community of oncology studies. These findings mirror FDA approvals over the past decade, with many approvals for additional indications in cancer types and treatments that already have approvals.^23^ The funding pattern is also consistent with the broader oncology trial community, with more than half of trials in the germline space receiving industry sponsorship and the remainder sponsored by academic, government, and cooperative group settings.^24^ Microsatellite instability status, often reflective of variations in high-penetrance Lynch syndrome genes (*MSH2, MSH6, MLH1, *and *PMS2*), estimates immunotherapy response.^25,26^ Yet any given mismatch repair gene was a biomarker in fewer than 20 studies, compared with more than 200 trials each for *BRCA1*/*2*. To expand the potential benefits of germline data in oncology across more patients, trials must expand beyond the well-trodden *BRCA*-PARP space.

Exciting steps toward broadening germline data use appear across a few domains. Pharmacogenomics was the most frequently studied pathway beyond DNA damage repair. Genes associated with pharmacogenomics are not typically included on hereditary cancer panels. Increasing data on variant penetrance, decreasing cost of testing, and inclusion of pharmacogenomic testing in guidelines (as with *UGT1A1* variants) offer practical reasons that pharmacogenomics can and should expand germline relevance in cancer care.^27^ The significant association with increased patient accrual we observed among trials that use germline eligibility criteria is encouraging for the prospect of expanding germline-associated studies. We also identified clinical trials designed to provide frameworks for studying germline modifiers of somatic biomarkers and treatment response (such as for *ERBB2* or *EGFR*) or identifying germline variants of known somatic biomarkers (as with *EGFR*). These trials and these patients will provide critical future ground for exploring how germline data influences cancer development and how somatic tumor data can guide future hereditary syndrome understanding.^28^

Our results validate and echo concerns about representation and bias in clinical trials, particularly regarding genetics. Oncology clinical trials using and collecting germline data have been conducted overwhelmingly in the US and Europe, reflecting existing global trends in oncology trials.^29^ In germline genetics, such trial patterns maintain existing global clinical trial and care access disparities and perpetuate existing disparities in genetics data availability across populations.^30,31,32,33^ Although our study found that such trials report higher-than-anticipated accrual rates, these rates do not necessarily translate to more diverse patient recruitment. Approximately 2% of patients in the US and European OlympiAD and OlympiA studies^34,35^ of Olaparib in metastatic and adjuvant breast cancer were self-reported Black race, compared with 13% of the US population. The FDA’s recent *Race and Ethnicity Diversity Plans* for trial development, as well as the National Cancer Institute’s Center for Global Health, established in the past decade, are initial steps to reverse these trends.^36,37^

The intersection of clinical trials and germline genetics underlay our interest in ascertaining to what extent inclusion and exclusion criteria may inadvertently exclude patients and contribute to research biases.^38,39,40^ Four trials did so overtly; 3^16,17,18^ mentioned Ashkenazi Jewish patients but conflated indications for germline testing and indications for cancer treatment, while 1^19^ stated that participating patients were assumed to have insurance coverage for genetic testing. Fifty-three trials required pathogenic germline variants without additional routes for trial inclusion, increasing the potential challenge for patients of color whose variants are more frequently classified as VUS due to lack of data.

A few strategies may counteract the biases we identified. At a minimum, ancestry-specific recruitment to clinical trials should be part of inclusion and exclusion criteria only when a study goal includes learning more about the relevant population or group. Although we would never advocate for enrolling all patients with germline VUSs in research studies without context, some clinical trials in our study highlighted alternative approaches. For instance, NCT04603365^41^ specified inclusion allowance for “[VUS] in patients with strong personal or family history where the clinician makes a presumed clinical diagnosis.” NCT03286842^42^ stated that “Mutations that are not clearly pathogenic may be assessed by a committee of genetic specialists to adjudicate if the patient is eligible.”

### Limitations

Our study has limitations aligned with Trialtrove data. Full Trialtrove data are available only via paid license. If fields in Trialtrove were insufficiently or inaccurately populated, we were not able to include those data in this work. We did not analyze distribution of individual-level race and ethnicity within trials, for instance. Trialtrove personnel were informed of errors we found to improve future annotation. Relative paucity of known outcome data, for example, is an overlapping weakness in both Trialtrove and ClinicialTrials.gov. We also did not have individual-level data, such as response rates, for meta-analyses of these trials.

There are 2 potential sources of error in our algorithm as related to Trialtrove. The Trialtrove curation method for adding genes to the Oncology Biomarker field for a trial is purely syntactic. However, Trialtrove curators may not capture biomarkers relevant to germline studies (if described generally as pharmacogenetic modifiers). This would result in undercounting we are not able to address. We may have also erroneously judged that a trial used germline data related to a biomarker when it did not. To limit overcounting, we manually reviewed and verified each trial for each biomarker associated with least 5 trials overall in eTable 2 in the Supplement.

## Conclusions

In the past decade, oncologists have dramatically advanced their clinical use of genetic data. Successes of targeted therapies developed from hereditary cancer genetics set the stage for future opportunities. This summary of clinical trials leveraging germline data provides an impetus for investigators and drug developers to think beyond *BRCA 1/2* and PARP inhibitors, to explore other targets from hereditary cancer syndromes, and to be more thoughtful about trial recruitment in the process.

## Notes

Supplement. **eAppendix. **Supplementary Methods
eReferences
**eTable 1.** Number and Percent of Clinical Trials (of 887 Total Trials Identified) That Study Germline Data for Each Cancer Type **eTable 2.** Biomarkers Associated With Trials With Germline Data in Trialtrove **eTable 3.** Mechanisms of Action of Drugs in Trials With Germline Data **eFigure 1.** End Points in Oncology Trials Using Germline Data for Eligibility vs. Trials Not Using Germline Data **eFigure 2.** Outcomes in Oncology Trials Using Germline Data for Eligibility vs. Trials Not Using Germline Data **eFigure 3.** Percent Accrual in Oncology Trials Using Germline Data for Eligibility vs. Trials Not Using Germline Data
