# Supplementary material for: Characterization of Oncology Clinical Trials Using Germline Genetic Data
Source: JAMA Netw Open. 2022 Nov 16;5(11):e2242370. doi: 10.1001/jamanetworkopen.2022.42370 (PMC9669814; doi:10.1001/jamanetworkopen.2022.42370)
Supplement: Supplement. — eAppendix. Supplementary Methods eReferences eTable 1. Number and Percent of Clinical Trials (of 887 Total Trials Identified) That Study Germline Data for Each Cancer Type eTable 2. Biomarkers Associated With Trials With Germline Data in Trialtrove eTable 3. Mechanisms of Action of Drugs in Trials With Germline Data eFigure 1. End Points in Oncology Trials Using Germline Data for Eligibility vs. Trials Not Using Germline Data eFigure 2. Outcomes in Oncology Trials Using Germline Data for Eligibility vs. Trials Not Using Germline Data eFigure 3. Percent Accrual in Oncology Trials Using Germline Data for Eligibility vs. Trials Not Using Germline Data [file jamanetwopen-e2242370-s001.pdf]

Supplemental Online Content

Kammula AV, Schäffer AA, Rajagopal PS. Characterization of oncology clinical trials using germline genetic data. *JAMA Netw Open*. 2022;5(11):e2242370. doi:10.1001/jamanetworkopen.2022.42370

eAppendix. Supplementary Methods
eReferences.
eTable 1. Number and Percent of Clinical Trials (of 887 Total Trials Identified) That Study Germline Data for Each Cancer Type
eTable 2. Biomarkers Associated With Trials With Germline Data in Trialtrove
eTable 3. Mechanisms of Action of Drugs in Trials With Germline Data
eFigure 1. End Points in Oncology Trials Using Germline Data for Eligibility vs. Trials Not Using Germline Data
eFigure 2. Outcomes in Oncology Trials Using Germline Data for Eligibility vs. Trials Not Using Germline Data
eFigure 3. Percent Accrual in Oncology Trials Using Germline Data for Eligibility vs. Trials Not Using Germline Data

This supplemental material has been provided by the authors to give readers additional information about their work.

eAppendix. Supplementary Methods

Search Strategy
We used Python version 3.8 to search nine Trialtrove columns entitled “Trial Title”, “Treatment Plan”, “Primary Tested Drug”, “Other Tested Drug”, “Primary Endpoint Details”, “Secondary/Other Endpoint Details”, “Trial Objective”, “Trial Results”, and “Trial Notes” for terms such as: “germline”, “hereditary”, “inherited.” We defined oncology trials as using germline information if any of the germline-related terms were present in the nine searched columns. We necessarily show any search terms with upper-case or lower-case letters in the text, but the actual searches with Python were case-insensitive.

For inclusion/exclusion criteria, we additionally searched the ‘Inclusion criteria’ and ‘Exclusion criteria” fields specifically for the above terms as well as for the terms “variant”, “mutation”, “allele”, and “pathogenic”. All candidate trials found by the automated search were reviewed manually and duplicates and false matches were removed, yielding n=322 trials.

Although only a subset of trials used germline data for inclusion/exclusion criteria, many trials are true positives for using germline information in the arm assignment or data analysis parts of the trial (e.g., analyzing whether the response is associated with some germline variant). This explains the total number of trials (n=887).

From Trialtrove, data were downloaded as Excel files. Using Python pandas (version 1.4.2), these files were converted into pandas DataFrames. Non-ASCII values, new line, and tab characters were removed. The standard re library (version 3.8) was used for searching the terms indicative of germline data use. The function re.search() was the primary method used from the re library. We then searched the structured Trialtrove columns: “Patient Segment”, “Disease”, "Trial Phase," “Trial Status,” Primary Endpoint,” “Trial Outcomes,” “Oncology Biomarker”, “Primary Tested Drug: Mechanism of Action”, “Primary Tested Drug, Therapeutic Class”, “Countries”, and “Sponsor Type”.

Analysis Note on “Patient Segments” in Trialtrove: The following segments were condensed for purpose of analysis: "stage iv", "metastatic," “unresectable,” “bone mets,” “cns mets,” “other mets,” “advanced,” “extensive” were condensed into “Stage IV/Advanced”; “Adjuvant” and “Neoadjuvant” were condensed into “Early Stage Not Otherwise Specified (NOS).”

Analysis Note on “Disease” in Trialtrove: For reporting purposes, cancer types were condensed or renamed into single categories as follows: Ovarian, primary peritoneal, and fallopian tube cancer into “Ovarian / Primary Peritoneal / Fallopian Tube”; “Unspecified solid tumor,” “Metastatic cancer,” “N/A,” “Unspecified cancer,” into “Other.” “Hematology: Other” included trials with focus on non-malignant diseases such as thalassemia, anemia and sickle-cell disease, and their association with cancers or cancer treatments as well as “unspecified hematologic cancers” and “transplantation/GVHD.” Cancer types were cross-checked when combined to avoid duplicate counts. See eTable 1 for full counts by Cancer Type.

Analysis Note on “Primary Endpoints” in Trialtrove: Primary Endpoints are taken directly from Trialtrove’s “Primary Endpoints” reporting. All trials had “Primary Endpoints” data. Consistent reporting was available for the following measures: “Safety and tolerability,” “dose-limiting toxicities,” “clinical benefit rate,” “response rate,” “disease-free survival,” “event-free survival,” “progression-free survival,” “time to progression,” “recurrence,” “overall survival,” as well as use of Common Terminology Criteria for Adverse Events (CTCAE) criteria and Response Evaluation Criteria in Solid Tumors (RECIST) criteria.

Analysis Note on “Outcomes” in Trialtrove: Outcomes were taken directly from Trialtrove’s “Trial Outcomes” reporting. As reported by Informa, a trial outcome is considered “positive” if “the trial's primary endpoint(s) were met with statistical significance and/or the trial's sponsor or investigator stated that it had a positive outcome or was successful.”[1] This designation corresponds to “Completed, Positive outcome/primary endpoint(s) met” or “Completed, Early positive outcome.” Where these were not available, Trial Status was used to infer the missing data. If Trial Status was denoted as Completed or Closed, this was listed as Completed or Closed – NOS.

We considered trials as having outcomes in Trialtrove if they were in any of the following categories:

- “Completed, Positive outcome/primary endpoint(s) met”

© 2022 Kammula AV et al. *JAMA Network Open.*

- “Completed, Early positive outcome”
- “Completed, Negative outcome/primary endpoint(s) not met”
- “Terminated, Poor enrollment”
- “Terminated, Lack of efficacy”
- “Terminated, Business decision” (all categories)
- “Terminated, Lack of funding”

We also included trials that had multiple reasons for Termination if one Termination reason was in the list above.

Analysis Note on “Biomarkers” in Trialtrove: Values in the Oncology Biomarker field are populated in Trialtrove when biomarkers or synonyms are present in one of eight other fields: Trial Title, Trial Objectives, Patient Population, Inclusion Criteria, Exclusion Criteria, Primary Endpoint Details, Secondary Endpoint Details, and Trial Results. Per Trialtrove, gene names in the biomarker column or synonyms are derived from NCBI Gene and CIVICmine.[2,3] Biomarkers included are those listed in Trialtrove wherein the gene or protein represented by that symbol is being actively evaluated in patients. We did not include biomarkers intended to represent general medical measures (such as albumin for nutritional status) or biomarkers that were not either genes or associated proteins except for homologous recombination deficiency (HRD).

To confirm biomarkers used by trials in Trialtrove, we completed four quality control procedures. First, we confirmed the frequency of biomarker values, including specifically parsing punctuation marks in the middle of biomarker sets (such as “*BRCA 1/2"* referring to *BRCA1* and *BRCA2*). Second, we verified that listed symbols were identifiable in the Human Genome Nomenclature Committee standard (genenames.org) and were identifiable on the list of valid gene symbol biomarkers in CivicMine, given its use as a source for Trialtrove.[3] Symbols not corresponding to gene names or not in CivicMine were removed. Third, we checked manually that mutations associated with gene symbols do correspond to those genes rather than acronyms for some other non-gene entity. These procedures helped clarify non-gene biomarkers in Trialtrove with the same name or symbol as a human gene, such as “event free survival” rather than the gene “embryonal Fyn-associated substrate” (*EFS*).

Fourth, we manually cross-checked each biomarker linked to at least 5 studies in Trialtrove with their corresponding trials and with ClinicalTrials.gov to confirm that the biomarker was, in fact, used in the trial and in a predictive or correlative context. We considered trials to have a germline-related association with the biomarker if (a) the trial expressly searched for germline modifiers of the somatic biomarker, (b) the trial studied germline mutations in the biomarker, or (c) the trial sought broad pharmacogenomic / germline associations and included patients classified by the somatic biomarker. We did not include trials that included the biomarker solely as part of exclusion criteria (such that germline data would not necessarily be collected as related to these patients), or if a trial was studying a therapy targeting the biomarker (such as an EGFR-targeting antibody) without any germline information use. The list of biomarkers can be found in eTable 2.

Analysis Note on “Treatments” in Trialtrove: For reporting purposes, treatments were condensed into single categories based on target family. For example, PARP inhibitors are condensed across families. "Angiogenesis” was condensed with VEGF inhibitors and VEGFR inhibitors. “Immuno-oncology therapy,” “immune checkpoint inhibitor,” “PD-1 antagonist,” “PD-L1 antagonist,” and “CTLA-4 antagonist” were condensed. “DNA inhibitor” and “DNA synthesis” were condensed. Combinations that were listed in the "Primary Tested Drug” are reported separately. The full list of Primary Tested Drugs and Primary Tested Combinations as in Trialtrove can be found in eTable 3.

© 2022 Kammula AV et al. *JAMA Network Open.*

eReferences

(1) Blazynski, C. Clinical Trial Landscape: 2015 Trial Completions [White paper]. Trialtrove Informa; 2016. Accessed September 21, 2022. https://pharmaintelligence.informa.com/~/media/informa-shop-window/pharma/files/pdfs/whitepapers/april-2016_trial-completions_christine-blazynski.pdf

(2) Brown GR, Hem V, Katz KS, et al. Gene: a gene-centered information resource at NCBI. *Nucleic Acids Res.* 2015;43(Database issue):D36-D42.

(3) Lever J, Jones MR, Danos AM, et al. Text-mining clinically relevant cancer biomarkers for curation into the CIViC database. *Genome Med.* 2019;11(1):78.

© 2022 Kammula AV et al. *JAMA Network Open.*

eTable 1. Number and Percent of Clinical Trials (of 887 Total Trials Identified) That Study Germline Data for Each Cancer Type.

Trials may enroll patients with more than one cancer type, so percentages do not correspond to 100%.

Cancer Type	Number of Trials	Percent of 887 Identified Trials
Oncology: Breast	259	29.2%
Oncology: Ovarian / Primary Peritoneal / Fallopian Tube[a]	168	18.9%
Oncology: Colorectal	112	12.6%
Oncology: Prostate	107	12.1%
Oncology: Lung, Non-Small Cell	99	11.2%
Oncology: Other[b]	87	9.8%
Oncology: Pancreas	78	8.8%
Oncology: Lymphoma, Non-Hodgkin's	67	7.6%
Oncology: Renal	60	6.8%
Oncology: Acute Lymphocytic Leukemia (ALL)	58	6.5%
Oncology: Gastro-Esophageal[c]	55	6.2%
Oncology: Soft Tissue Sarcoma	52	5.9%
Oncology: Head/Neck	48	5.4%
Oncology: Acute Myelogenous Leukemia (AML)	47	5.3%
Oncology: Melanoma	40	4.5%
Oncology: Endometrial	35	4.0%
Oncology: Neuroendocrine	34	3.8%
Oncology: Myelodysplastic Syndrome	33	3.7%
Oncology: Bladder	32	3.6%
Oncology: Liver	30	3.4%
Oncology: Chronic Lymphocytic Leukemia (CLL)	24	2.7%
Oncology: Thyroid	21	2.4%
Oncology: Lung, Small Cell	21	2.4%
Oncology: Cervical	21	2.4%

© 2022 Kammula AV et al. *JAMA Network Open.*

Hematology: Other[d]	19	2.1%
**Cancer Type**	**Number of Trials**	**Percent of 887 Identified Trials**
Oncology: CNS, Glioblastoma	19	2.1%
Oncology: Mesothelioma	18	2.0%
Oncology: Bile Duct (Cholangiocarcinoma) / Gallbladder[e]	18	2.0%
Oncology: Lymphoma, Hodgkin's	16	1.8%
Oncology: Multiple Myeloma	16	1.8%
Oncology: Chronic Myelogenous Leukemia (CML)	14	1.6%
Oncology: Osteosarcoma	13	1.5%
Oncology: CNS, Other	13	1.5%
Oncology: CNS, Medulloblastoma	13	1.5%
Oncology: Myeloproliferative Neoplasms	11	1.2%
Oncology: GIST	10	1.1%
Oncology: Neuroblastoma	8	0.9%
Oncology: Testicular	7	0.8%
Oncology: Skin, Basal Cell Carcinoma	6	0.7%
Oncology: Supportive Care[f]	6	0.7%
Oncology: Anal	2	0.2%
Oncology: Skin, Squamous Cell Carcinoma (cSCC)	2	0.2%
Oncology: Vaginal	1	0.1%
(a) Category includes Oncology: Ovarian (167 trials), Oncology: Primary Peritoneal (112 trials), and Oncology: Fallopian Tube (111 trials)		
(b) Category includes Oncology: Unspecified solid tumor (61 trials), Oncology: Metastatic Cancer (21 trials), Oncology: N/A (12 trials), and Oncology: Unspecified cancer (7 trials)		
(c) Category includes Oncology: Gastric (47 trials) and Oncology: Esophageal (37 trials)		
(d) Category includes: Transplant/GVHD (11 trials), Oncology: Unspecified Hematologic cancer (7 trials), Thalassemia (4 trials), Anemia (2 trials), Sickle Cell Disease (2 trials)		
(e) Category includes Oncology: Bile Duct (17 trials) and Oncology: Gallbladder (7 trials)		
(f) Supportive care trials are listed as follows:		
NCT00255606: A Phase III Trial Comparing Docetaxel Every Third Week to Biweekly Docetaxel Monotherapy in Metastatic Hormone Refractory Prostate Cancer Patients - PROSTY Trial		

© 2022 Kammula AV et al. *JAMA Network Open.*

NCT02311907: The Use of Glutathione (GSH) for Prevention of Paclitaxel/Carboplatin (TAXOL/CBDCA) Induced Peripheral Neuropathy: A Phase III Randomized, Double-Blind Placebo Controlled Study
(NCT Not available) Association of ABCB1, 5-HT3B Receptor and CYP2D6 Genetic Polymorphisms with Ondansetron and Metoclopramide Antiemetic Response in Indonesian Cancer Patients Treated with Highly Emetogenic Chemotherapy
NCT02125344: A Randomized Phase III Trial Comparing Two Dose-dense, Dose-intensified Approaches (ETC and PM(Cb)) for Neoadjuvant Treatment of Patients With High-risk Early Breast Cancer (GeparOcto)
(NCT Not available) Randomized Phase II CAbazitaxel Dose Individualization and Neutropenia Prevention TriAl (CAINTA)
NCT04001829: Prospective Validation Trial of Taxane Therapy (Docetaxel or Weekly Paclitaxel) and Risk of Chemotherapy-Induced Peripheral Neuropathy in African American Women

© 2022 Kammula AV et al. *JAMA Network Open.*

eTable 2. Biomarkers Associated With Trials With Germline Data in Trialtrove.

Trials may collect multiple biomarkers. Biomarkers are not clearly differentiated in Trialtrove between gene and protein; as such, overlapping biomarkers (such as mutations in a gene) are consolidated. Trials that test for a gene in general and for specific mutations are counted exactly once for the gene total. Corresponding pathways are obtained from the Gene Ontology: Biological Process pathway set.

Biomarker	Number of Trials	Pathway
BRCA2	228	DNA Repair
BRCA1	224	DNA Repair
HRD[a]	80	Other
PALB2	59	DNA Repair
ATM	55	DNA Repair
ERBB2	44	Signal Transduction
CHEK2	42	Serine/Threonine Kinase Activity
EGFR	40	Signal Transduction
RAD51C	31	DNA Repair
BRIP1	30	DNA Repair
RAD51D	28	DNA Repair
BARD1	22	Apoptotic Process
MSH2	19	DNA Mismatch Repair
ABCB1	18	Transmembrane Transport
CDK12	18	Serine/Threonine Kinase Activity
NBN	18	DNA Repair
TP53	17	Apoptotic Process
PTEN	17	De-Phosphorylation
FANCA	17	DNA Repair
RAD51	16	DNA Repair
MLH1	15	DNA Mismatch Repair
MRE11	15	DNA Repair
CHEK1	14	Serine/Threonine Kinase Activity
ATR	13	Serine/Threonine Kinase Activity
MSH6	13	DNA Mismatch Repair
RAD51B	13	DNA Repair
FANCL	12	DNA Repair
XRCC1	11	Base-Excision Repair
RAD50	11	DNA Repair
ERCC2	11	Base-Excision Repair
VEGFA	10	Angiogenesis
PMS2	10	DNA Mismatch Repair
UGT1A6	10	Pharmacogenetics

© 2022 Kammula AV et al. *JAMA Network Open.*

UGT1A1	10	Pharmacogenetics
UGT1A7	10	Pharmacogenetics
UGT1A8	10	Pharmacogenetics
HLA-DRB1	10	Immune Response
MTHFR	10	Metabolism
CYP3A4	9	Pharmacogenetics
DPYD	9	Nucleotides
ARID1A	9	Chromatin Remodeling
ERCC1	9	Base-excision Repair
BLM	9	DNA Replication
FANCC	9	DNA Repair
RAD54L	9	DNA Repair
CYP2D6	8	Pharmacogenetics
BAP1	8	Erythrocyte Maturation
GSTP1	8	Metabolism
FANCM	7	DNA Repair
FCGR3A	7	Immune Response
FCGR2A	7	Immune Response
PPP2R2A	7	Serine/Threonine Kinase Activity
KMT2A (MLL)	6	Histone Modification
CYP3A5	6	Pharmacogenetics
RET	6	Phosphorylation
FANCD2	6	DNA Repair
FANCF	6	DNA Repair
FANCG	6	DNA Repair
EGF	5	Signal Transduction
VHL	5	Other
NF1	5	Signal Transduction
ABRAXAS1	5	DNA Repair
WRN	5	DNA Replication
CYP2B6	4	Pharmacogenetics
KDR	4	Angiogenesis
CCND1	4	Wnt Signaling
CYP2C8	4	Pharmacogenetics
DDX41	4	RNA Helicase
CYP2A6	4	Pharmacogenetics
PARP1	3	DNA ADP-Ribosylation
PIK3CA	3	Phosphatidylinositol-mediated Signaling
PIK3CB	3	Phosphatidylinositol-Mediated Signaling

© 2022 Kammula AV et al. *JAMA Network Open.*

PIK3CD	3	Phosphatidylinositol-Mediated Signaling
PIK3CG	3	Phosphatidylinositol-Mediated Signaling
CYP1A2	3	Pharmacogenetics
SLCO1B1/3	3	Transmembrane Transport
ATRX	3	Chromatin Remodeling
EPCAM	3	Transmembrane Transport
STK11	3	Serine/Threonine Kinase Activity
BRD4	3	Chromatin Remodeling
KRAS	2	Ras Protein Signal Transduction
RB1	2	Cell Cycle
ABCG2	2	Transmembrane Transport
IDH1	2	Metabolism
IDH2	2	Metabolism
CCNE1	2	Cell Cycle
PGR	1	Hormone Activity
BRAF	1	Signal Transduction
BCR	1	GTPase Regulation
CDKN2A (p16)	1	Serine/Threonine Kinase Activity
RRM1	1	DNA Replication
TNF	1	Immune Response
CDK6	1	Serine/Threonine Kinase Activity
IGH	1	Immune Response
CALR	1	Protein Structure
CEBPA	1	DNA Transcription
ESR1	1	Hormone Activity
AKT1	1	Serine/Threonine Kinase Activity
PDGFRB	1	Platelet-Derived Growth Factor Receptor Signaling
BCL2	1	Apoptotic Process
NOTCH1	1	NOTCH Pathway
CXCL8	1	Immune Response
AFF1	1	DNA Transcription
CUX1	1	Other
FGF2	1	FGFR Pathway
SMARCA4	1	DNA Transcription
TERT	1	Telomeres

(a) HRD is short for homologous repair deficiency and represents a set of genes tested for mutations; the exact set of HRD genes tested is not necessarily identical among the 80 trials

© 2022 Kammula AV et al. *JAMA Network Open.*

eTable 3. Mechanisms of Action of Drugs in Trials With Germline Data.
Each mechanism is counted across the 887 trials that use germline information in “Primary Tested Drug: Mechanism of Action,” “Number of Trials,” and “Percent of 887 Identified Trials.” Therapies are aggregated by target family; collapsed categories are noted with “/”. However, combinations of drugs that were listed as the “Primary Tested Drug” are maintained as documented in Trialtrove in columns “Primary Tested Combination of Drugs: Mechanisms of Action” and “Number of Trials.”

Primary Tested Drug: Mechanism of Action	Number of Trials	Percent of 887 Identified Trials
Poly ADP ribose polymerase inhibitor (1/2/3)	367	41.4%
Angiogenesis / VEGF / VEGFR inhibitors	331	37.3%
Immuno-oncology therapy / Immune checkpoint inhibitor / PD-1 antagonist / PD-L1 antagonist / CTLA-4 antagonist	253	28.5%
DNA repair enzyme inhibitor	103	11.6%
DNA inhibitor / DNA synthesis inhibitor	100	11.3%
DNA topoisomerase I/II inhibitors	68	7.7%
EGFR kinase inhibitor / antagonist	63	7.1%
FGFR inhibitors (1/2/3/4)	58	6.5%
c-KIT inhibitor	57	6.4%
Thymidylate synthase inhibitor	56	6.3%
*Platelet-derived growth factor receptor (PDGFR) kinase inhibitors (alpha / beta)	56	6.3%
Immunosuppressant	55	6.2%
Microtubule stimulant	46	5.2%
RET tyrosine kinase inhibitor	40	4.5%
Radical formation stimulant	30	3.4%
Apoptosis stimulant	30	3.4%
Dihydrofolate reductase inhibitor	29	3.3%
FLT-3 antagonist	27	3.0%
Tubulin inhibitor	27	3.0%
ERBB-2 tyrosine kinase inhibitor / antagonist / ERBB-4 / ERBB-3	27	3.0%
Taxane	23	2.6%

© 2022 Kammula AV et al. *JAMA Network Open.*

Cell cycle inhibitor	22	2.5%
**Primary Tested Drug: Mechanism of Action**	**Number of Trials**	**Percent of 887 Identified Trials**
Protein kinase inhibitor	21	2.4%
Tyrosine kinase inhibitor (TKI) (Not specified)	19	2.1%
RNA synthesis inhibitor	19	2.1%
Estrogen receptor antagonist	18	2.0%
B-RAF kinase inhibitor	17	1.9%
T cell stimulant	17	1.9%
Other	17	1.9%
Cyclin-dependent kinase inhibitors (6/4/9/2/7)	17	1.9%
mTOR kinase inhibitor	14	1.6%
Immunostimulant	14	1.6%
PI3 kinase inhibitor (alpha, beta, gamma, delta)	14	1.6%
MET tyrosine kinase inhibitor	13	1.5%
Asparaginase stimulant	12	1.4%
Colony stimulating factor 1 receptor antagonist	12	1.4%
BCR-ABL inhibitor	12	1.4%
Beta tubulin inhibitor	11	1.2%
Ribonucleoside diphosphate reductase inhibitor	11	1.2%
RAF kinase inhibitor	10	1.1%
EPHA receptor kinase inhibitor	10	1.1%
DNA directed DNA polymerase inhibitor	9	1.0%
Aromatase inhibitor	9	1.0%
Epidermal growth factor antagonist	8	0.9%
Protein kinase B inhibitor	8	0.9%
Platelet aggregation inhibitor	8	0.9%
Steroid synthesis inhibitor	8	0.9%

© 2022 Kammula AV et al. *JAMA Network Open.*

Prostaglandin synthase inhibitor	8	0.9%
c-RAF kinase inhibitor	7	0.8%
**Primary Tested Drug: Mechanism of Action**	**Number of Trials**	**Percent of 887 Identified Trials**
CD20 antagonist	7	0.8%
AXL receptor tyrosine kinase inhibitor	7	0.8%
Microtubule inhibitor	7	0.8%
17,20 lyase inhibitor	7	0.8%
Ataxia telangiectasia RAD3 inhibitor	7	0.8%
Cyclooxygenase inhibitor	7	0.8%
Thromboxane synthase inhibitor	7	0.8%
Lymphocyte-specific tyrosine kinase inhibitor	7	0.8%
SRC inhibitor	7	0.8%
FYN tyrosine kinase inhibitor	7	0.8%
Radiopharmaceutical	7	0.8%
Selective estrogen receptor modulator	6	0.7%
Selective estrogen receptor downregulator	6	0.7%
Transcription factor NF-kappaB inhibitor	6	0.7%
Cereblon E3 ubiquitin ligase stimulant	6	0.7%
Protein degrader	5	0.6%
Disease modifying antirheumatic drug	5	0.6%
Androgen receptor antagonist	5	0.6%
ROS receptor tyrosine kinase inhibitor	5	0.6%
Bruton tyrosine kinase inhibitor	5	0.6%
Cyclooxygenase 2 inhibitor	4	0.5%
p53 stimulant	4	0.5%
Proteasome inhibitor	4	0.5%
Osteoclast inhibitor	4	0.5%

© 2022 Kammula AV et al. *JAMA Network Open.*

Bone resorption inhibitor	4	0.5%
TIE-2 tyrosine kinase inhibitor	4	0.5%
Histone deacetylase inhibitor	4	0.5%
**Primary Tested Drug: Mechanism of Action**	**Number of Trials**	**Percent of 887 Identified Trials**
Protein kinase C beta inhibitor	4	0.5%
Insulin-like growth factor 1 antagonist	4	0.5%
Ribonucleoside triphosphate reductase inhibitor	4	0.5%
Hedgehog pathway inhibitor	4	0.5%
Anaplastic lymphoma kinase inhibitor	4	0.5%
Ribosomal S6 kinase inhibitor	4	0.5%
Mitogen-activated protein kinase kinase 2 inhibitor	4	0.5%
T cell inhibitor	4	0.5%
Somatostatin receptor agonist (5/1/2/3)	4	0.5%
Bisphosphonate	3	0.3%
Vinca alkaloid	3	0.3%
Heat shock protein 90 antagonist	3	0.3%
Phosphoribosylglycinamide formyltransferase inhibitor	3	0.3%
Reducing agent	3	0.3%
Orotate phosphoribosyltransferase inhibitor	3	0.3%
Janus kinase 2 inhibitor	3	0.3%
TRKA tyrosine kinase inhibitor	3	0.3%
Calcineurin inhibitor	3	0.3%
Protein synthesis inhibitor	3	0.3%
RNA polymerase inhibitor	3	0.3%
Interferon alpha 2b agonist	3	0.3%
DNA methylase inhibitor	3	0.3%
TRKB tyrosine kinase inhibitor	3	0.3%

© 2022 Kammula AV et al. *JAMA Network Open.*

Mitogen-activated protein kinase kinase 1 inhibitor	3	0.3%
Toll-like receptor agonist (8/7/3)	3	0.3%
Bromodomain containing inhibitor (4/2/3)	3	0.3%
Epithelial cell adhesion molecule inhibitor	2	0.2%
**Primary Tested Drug: Mechanism of Action**	**Number of Trials**	**Percent of 887 Identified Trials**
Glucocorticoid agonist	2	0.2%
Toll-like receptor 9 agonist	2	0.2%
HMGcoA reductase inhibitor	2	0.2%
ABL receptor tyrosine kinase inhibitor	2	0.2%
Dihydropyrimidine dehydrogenase inhibitor	2	0.2%
Adenosine deaminase inhibitor	2	0.2%
Progesterone receptor agonist	2	0.2%
Angiopoietin inhibitor	2	0.2%
RANKL antagonist	2	0.2%
Checkpoint kinase 1 inhibitor	2	0.2%
Checkpoint kinase 2 inhibitor	2	0.2%
TRKC tyrosine kinase inhibitor	2	0.2%
G-quadruplex stabiliser	2	0.2%
LYN tyrosine kinase inhibitor	2	0.2%
CD19 antagonist	2	0.2%
Lysine (K)-specific demethylase 1A inhibitor	2	0.2%
MDM2 inhibitor	2	0.2%
Hepcidin stimulant	2	0.2%
Hypoxia-inducible factor 2 alpha antagonist	2	0.2%
Gene expression inhibitor	2	0.2%
IAP antagonist	2	0.2%
Granulocyte colony stimulating factor agonist	2	0.2%

© 2022 Kammula AV et al. *JAMA Network Open.*

Transmembrane protease, serine 6 inhibitor	2	0.2%
Interleukin 8 antagonist	1	0.1%
Calcium channel antagonist	1	0.1%
Cysteine protease stimulant	1	0.1%
Kinesin inhibitor	1	0.1%
**Primary Tested Drug: Mechanism of Action**	**Number of Trials**	**Percent of 887 Identified Trials**
H+ K+ transporting ATPase inhibitor	1	0.1%
MTORC2 kinase inhibitor	1	0.1%
MTORC1 kinase inhibitor	1	0.1%
CD52 antagonist	1	0.1%
Free radical scavenger	1	0.1%
Telomerase stimulant	1	0.1%
Flavinoid agonist	1	0.1%
Heparin-binding EGF-like growth factor antagonist	1	0.1%
CD22 antagonist	1	0.1%
Hepatocyte growth factor receptor antagonist	1	0.1%
B-cell activating factor inhibitor	1	0.1%
Secretase gamma inhibitor	1	0.1%
Glutaminase inhibitor	1	0.1%
Iron absorption stimulant	1	0.1%
Enhancer of zeste homolog 2 inhibitor	1	0.1%
CD3 agonist	1	0.1%
Inhibin, beta A inhibitor	1	0.1%
Activin receptor type II antagonist	1	0.1%
Protein kinase C inhibitor	1	0.1%
Cyclooxygenase 1 inhibitor	1	0.1%
Granulocyte macrophage colony stimulating factor agonist	1	0.1%

© 2022 Kammula AV et al. *JAMA Network Open.*

Protein 50S ribosomal subunit inhibitor	1	0.1%
Gluconeogenesis inhibitor	1	0.1%
Biguanide	1	0.1%
Dihydroorotate dehydrogenase inhibitor	1	0.1%
CXC chemokine receptor 4 antagonist	1	0.1%
YES tyrosine kinase inhibitor	1	0.1%
**Primary Tested Drug: Mechanism of Action**	**Number of Trials**	**Percent of 887 Identified Trials**
Luteinizing hormone releasing hormone (LHRH) antagonist	1	0.1%
Follicle-stimulating hormone receptor antagonist	1	0.1%
SYK tyrosine kinase inhibitor	1	0.1%
Vitamin D agonist	1	0.1%
Cholesterol side-chain cleavage enzyme inhibitor	1	0.1%
Phosphodiesterase 4 inhibitor	1	0.1%
Wee-1 tyrosine kinase inhibitor	1	0.1%
MDM4 inhibitor	1	0.1%
Immune checkpoint stimulant	1	0.1%
Vitamin C agonist	1	0.1%
Genome editing	1	0.1%
CD47 antagonist	1	0.1%
Hypoxia-inducible factor 1 alpha antagonist	1	0.1%
Janus kinase inhibitor	1	0.1%
KIR-mediated natural killer cell inhibition antagonist	1	0.1%
Farnesyltransferase inhibitor	1	0.1%

Primary Tested Combination of Drugs: Mechanisms of Action	Number of Trials
DNA inhibitor / DNA synthesis inhibitor	41
DNA inhibitor / DNA synthesis inhibitor / immunosuppressant	15

© 2022 Kammula AV et al. *JAMA Network Open.*

Disease modifying antirheumatic drug / immunosuppressant	13
Corticosteroid agonist / microtubule inhibitor	12
Corticosteroid agonist / DNA synthesis inhibitor	11
Poly ADP ribose polymerase 1 inhibitor / PD-L1 antagonist	10
DNA synthesis inhibitor / immunosuppressant	9
Taxane / DNA inhibitor	9
**Primary Tested Combination of Drugs: Mechanisms of Action**	**Number of Trials**
Angiogenesis inhibitor / poly ADP ribose polymerase 2 inhibitor	9
Poly ADP ribose polymerase 1 inhibitor / PD-1 antagonist	9
RNA synthesis inhibitor / DNA inhibitor	9
RNA synthesis inhibitor / immunosuppressant	8
RNA synthesis inhibitor / beta tubulin inhibitor	8
RNA synthesis inhibitor / unidentified pharmacological activity	8
Immune checkpoint inhibitor / CTLA4 antagonist	8
RNA synthesis inhibitor / DNA synthesis inhibitor	7
Immune checkpoint inhibitor / poly ADP ribose polymerase 2 inhibitor	7
Cell cycle inhibitor / immunosuppressant	6
Apoptosis stimulant / immunosuppressant	6
Cell cycle inhibitor / DNA inhibitor	6
DNA inhibitor / poly ADP ribose polymerase 2 inhibitor	6
Immunostimulant / T cell stimulant	6
Disease modifying antirheumatic drug / protein synthesis inhibitor	5
Poly ADP ribose polymerase 1 inhibitor / DNA inhibitor	5
Poly ADP ribose polymerase 1 inhibitor / cyclin-dependent kinase 4 inhibitor	5
Vinca alkaloid / DNA synthesis inhibitor	5
Corticosteroid agonist / DNA inhibitor	4
Disease modifying antirheumatic drug / microtubule inhibitor	4

© 2022 Kammula AV et al. *JAMA Network Open.*

17,20 lyase inhibitor / androgen receptor antagonist	4
Taxane / DNA synthesis inhibitor	4
RNA synthesis inhibitor / microtubule inhibitor	4
Immune checkpoint inhibitor / PD-L1 antagonist	4
DNA inhibitor / immunosuppressant	3
DNA synthesis inhibitor / beta tubulin inhibitor	3
Cell cycle inhibitor / DNA synthesis inhibitor	3
**Primary Tested Combination of Drugs: Mechanisms of Action**	**Number of Trials**
DNA repair enzyme inhibitor / immunosuppressant	3
Corticosteroid agonist /immunosuppressant	3
Vinca alkaloid / unidentified pharmacological activity / DNA synthesis inhibitor	3
EGFR antagonist / VEGFR antagonist	3
RNA synthesis inhibitor / radical formation stimulant	3
Apoptosis stimulant / arachidonic acid inhibitor	3
Protein degrader / immunosuppressant	3
Vinca alkaloid / asparaginase stimulant / DNA synthesis inhibitor	3
Vinca alkaloid / immunosuppressant	3
Poly ADP ribose polymerase 1 inhibitor / poly ADP ribose polymerase 2 inhibitor	3
Tyrosine kinase inhibitor (TKI) / immunosuppressant	3
Angiogenesis inhibitor / immunosuppressant	3
Corticosteroid agonist / steroid synthesis inhibitor	3
Protein degrader / estrogen receptor antagonist	3
Taxane / poly ADP ribose polymerase 2 inhibitor	3
Aromatase inhibitor / cyclin-dependent kinase 4 inhibitor	3
Immune checkpoint inhibitor / B-RAF kinase inhibitor	3
MET tyrosine kinase inhibitor / PD-1 antagonist	3
Cell cycle inhibitor / poly ADP ribose polymerase 2 inhibitor	3

© 2022 Kammula AV et al. *JAMA Network Open.*

YES tyrosine kinase inhibitor / ABL receptor / tyrosine kinase inhibitor	3
Protein kinase inhibitor / mitogen-activated protein kinase kinase 1 inhibitor	3
17,20 lyase inhibitor / poly ADP ribose polymerase 2 inhibitor	3
RNA synthesis inhibitor / DNA synthesis inhibitor / immunosuppressant	2
Corticosteroid agonist / CD20 antagonist / microtubule inhibitor	2
Aromatase inhibitor / estrogen receptor antagonist	2
DNA topoisomerase i inhibitor / DNA inhibitor / DNA synthesis inhibitor	2
Cell cycle inhibitor / beta tubulin inhibitor	2
**Primary Tested Combination of Drugs: Mechanisms of Action**	**Number of Trials**
Poly ADP ribose polymerase 3 inhibitor / poly ADP ribose polymerase 2 inhibitor	2
Vinca alkaloid / unidentified pharmacological activity / DNA inhibitor / asparaginase stimulant / DNA synthesis inhibitor	2
Proteasome inhibitor / angiogenesis inhibitor	2
Disease modifying antirheumatic drug / unidentified pharmacological activity / immunosuppressant	2
RNA synthesis inhibitor / DNA synthesis inhibitor / microtubule inhibitor	2
Tyrosine kinase inhibitor (TKI) / EGFR kinase inhibitor	2
Cell cycle inhibitor / VEGFR antagonist	2
Apoptosis stimulant / DNA synthesis inhibitor	2
DNA topoisomerase i inhibitor / poly ADP ribose polymerase 2 inhibitor	2
RNA synthesis inhibitor / unidentified pharmacological activity / DNA inhibitor	2
Tyrosine kinase inhibitor (TKI) / VEGFR antagonist	2
Cell cycle inhibitor / DNA inhibitor	2
Proteasome inhibitor / transcription factor nf-kappab inhibitor	2
Poly ADP ribose polymerase 1 inhibitor / VEGFR antagonist	2
EGFR antagonist / DNA topoisomerase i inhibitor	2
Aromatase inhibitor / selective estrogen receptor downregulator	2
Immune checkpoint inhibitor / mitogen-activated protein kinase kinase 1 inhibitor	2
ERBB-2 tyrosine kinase inhibitor / FGFR antagonist	2

© 2022 Kammula AV et al. *JAMA Network Open.*

MTORC2 kinase inhibitor / protein kinase B inhibitor	2
Mitogen-activated protein kinase kinase 2 inhibitor / mtorc1 kinase inhibitor	2
Apoptosis stimulant / LYN tyrosine kinase inhibitor	2
Angiogenesis inhibitor / anaplastic lymphoma kinase inhibitor	2
Angiogenesis inhibitor/ c-KIT inhibitor	2
Angiogenesis inhibitor / LYN tyrosine kinase inhibitor	2
Immune checkpoint inhibitor / immunosuppressant	2
RNA synthesis inhibitor / transcription factor nf-kappab inhibitor	2
Mitogen-activated protein kinase kinase 2 inhibitor / poly ADP ribose polymerase 2 inhibitor	2
**Primary Tested Combination of Drugs: Mechanisms of Action**	**Number of Trials**
Immune checkpoint inhibitor / immunostimulant	2
Immune checkpoint inhibitor / VEGFR antagonist	2
Protein degrader / poly ADP ribose polymerase 2 inhibitor	2
Taxane / androgen receptor antagonist	2
Poly ADP ribose polymerase 1 inhibitor / ataxia telangiectasia RAD3 inhibitor	2
Poly ADP ribose polymerase 1 inhibitor / FGFR1 tyrosine kinase inhibitor	2
RNA synthesis inhibitor / DNA inhibitor / beta tubulin inhibitor	1
Vinca alkaloid / unidentified pharmacological activity / asparaginase stimulant / DNA synthesis inhibitor	1
Androgen receptor antagonist / luteinizing hormone releasing hormone (LHRH) agonist	1
DNA inhibitor / unidentified pharmacological activity	1
DNA synthesis inhibitor / microtubule inhibitor	1
DNA topoisomerase ii inhibitor / radical formation stimulant	1
T cell stimulant / immunosuppressant	1
Disease modifying antirheumatic drug / DNA inhibitor / asparaginase stimulant / radical formation stimulant	1
DNA repair enzyme inhibitor / interleukin 2 agonist	1
Cell cycle inhibitor / ribonucleoside diphosphate reductase inhibitor	1
Taxane / unidentified pharmacological activity	1

© 2022 Kammula AV et al. *JAMA Network Open.*

DNA topoisomerase I inhibitor / DNA inhibitor	1
DNA topoisomerase II inhibitor / CD20 antagonist	1
Disease modifying antirheumatic drug / corticosteroid agonist / immunosuppressant	1
Corticosteroid agonist / unidentified pharmacological activity / immunosuppressant	1
Vinca alkaloid / RNA synthesis inhibitor / DNA inhibitor	1
Cell cycle inhibitor / microtubule inhibitor	1
Bone resorption inhibitor / osteoclast inhibitor	1
RNA synthesis inhibitor / BCR-ABL inhibitor	1
Apoptosis stimulant / unidentified pharmacological activity / protein synthesis inhibitor	1
Vinca alkaloid / DNA inhibitor / microtubule inhibitor	1
**Primary Tested Combination of Drugs: Mechanisms of Action**	**Number of Trials**
DNA topoisomerase ii inhibitor / DNA synthesis inhibitor	1
RNA synthesis inhibitor / DNA topoisomerase ii inhibitor	1
DNA repair enzyme inhibitor / granulocyte colony stimulating factor agonist / DNA synthesis inhibitor	1
RNA synthesis inhibitor / EGFR antagonist / DNA topoisomerase i inhibitor	1
Bone resorption inhibitor / cyclooxygenase 2 inhibitor / steroid synthesis inhibitor	1
Taxane / osteoclast inhibitor	1
Corticosteroid agonist / arachidonic acid inhibitor	1
RNA synthesis inhibitor / DNA inhibitor / unidentified pharmacological activity	1
RNA synthesis inhibitor / DNA synthesis inhibitor / DNA inhibitor	1
DNA synthesis inhibitor / asparaginase stimulant	1
EGFR antagonist / DNA topoisomerase I inhibitor/ DNA inhibitor	1
Selective estrogen receptor modulator / testosterone receptor agonist	1
DNA synthesis inhibitor / DNA topoisomerase II inhibitor	1
PI3 kinase inhibitor / MTORC1 kinase inhibitor	1
Cell cycle inhibitor / unidentified pharmacological activity	1
Isoflavone agonist / thromboxane synthase inhibitor	1

© 2022 Kammula AV et al. *JAMA Network Open.*

Dihydropyrimidine dehydrogenase inhibitor / DNA topoisomerase I inhibitor / DNA inhibitor	1
DNA repair enzyme inhibitor /granulocyte colony stimulating factor agonist	1
Tyrosine kinase inhibitor (TKI) / DNA topoisomerase I inhibitor / DNA inhibitor	1
Cell cycle inhibitor / granulocyte colony stimulating factor agonist / microtubule inhibitor	1
Disease modifying antirheumatic drug / granulocyte colony stimulating factor agonist / DNA inhibitor	1
Vinca alkaloid / microtubule inhibitor	1
Vinca alkaloid / unidentified pharmacological activity / DNA topoisomerase I inhibitor immunosuppressant	1
T cell inhibitor / protein synthesis inhibitor	1
Disease modifying antirheumatic drug / DNA inhibitor	1
Disease modifying antirheumatic drug / corticosteroid agonist / immunosuppressant	1
RNA synthesis inhibitor / corticosteroid agonist / DNA synthesis inhibitor / immunosuppressant	1
**Primary Tested Combination of Drugs: Mechanisms of Action**	**Number of Trials**
Glucocorticoid agonist / immunosuppressant	1
DNA topoisomerase ii inhibitor / immunosuppressant	1
Corticosteroid agonist DNA inhibitor / unidentified pharmacological activity / immunosuppressant	1
RNA synthesis inhibitor / DNA synthesis inhibitor / protein synthesis inhibitor	1
Proteasome inhibitor / DNA inhibitor	1
Vinca alkaloid / asparaginase stimulant	1
Protein degrader / DNA inhibitor	1
Corticosteroid agonist / angiogenesis inhibitor	1
Glucocorticoid agonist / immunosuppressant	1
Mitotic inhibitor DNA synthesis inhibitor / immunosuppressant	1
Vinca alkaloid / immunosuppressant	1
Mitotic inhibitor / DNA synthesis inhibitor / microtubule inhibitor	1
RNA synthesis inhibitor / DNA synthesis inhibitor / corticosteroid agonist / immunosuppressant	1
Corticosteroid agonist / asparaginase stimulant	1
Vinca alkaloid / unidentified pharmacological activity / DNA synthesis inhibitor / corticosteroid agonist / immunosuppressant	1

© 2022 Kammula AV et al. *JAMA Network Open.*

RNA synthesis inhibitor / EGFR antagonist / DNA inhibitor / unidentified pharmacological activity	1
Disease modifying antirheumatic drug / VEGFR antagonist	1
Progesterone receptor agonist / estrogen receptor agonist	1
Immunostimulant / telomerase stimulant	1
DNA topoisomerase II inhibitor / CD20 antagonist / DNA synthesis inhibitor	1
Proteasome inhibitor / RET tyrosine kinase inhibitor	1
RNA synthesis inhibitor / DNA topoisomerase I inhibitor / DNA inhibitor	1
MET tyrosine kinase inhibitor / anaplastic lymphoma kinase inhibitor	1
DNA repair enzyme inhibitor / radical formation stimulant	1
Taxane DNA inhibitor / beta tubulin inhibitor	1
DNA repair enzyme inhibitor / protein synthesis inhibitor	1
Corticosteroid agonist / protease/peptidase inhibitor	1
**Primary Tested Combination of Drugs: Mechanisms of Action**	**Number of Trials**
Protein degrader / angiogenesis inhibitor	1
Protein degrader / protease/peptidase inhibitor	1
Angiogenesis inhibitor / DNA inhibitor	1
RNA synthesis inhibitor / poly ADP ribose polymerase 2 inhibitor	1
Taxane DNA inhibitor / DNA synthesis inhibitor	1
DNA topoisomerase I inhibitor / tubulin inhibitor	1
Aromatase inhibitor / janus kinase 1 inhibitor	1
Mitogen-activated protein kinase kinase 2 inhibitor / MET tyrosine kinase inhibitor / PI3 kinase alpha inhibitor / anaplastic lymphoma kinase inhibitor	1
PI3 kinase gamma inhibitor / PI3 kinase alpha inhibitor	1
Poly ADP ribose polymerase 1 inhibitor / protein kinase inhibitor	1
RNA synthesis inhibitor / DNA topoisomerase II inhibitor / VEGFR antagonist	1
Tubulin inhibitor / poly ADP ribose polymerase 2 inhibitor	1
RNA synthesis inhibitor / unidentified pharmacological activity / VEGFR antagonist	1
DNA inhibitor / DNA methylase inhibitor / granulocyte macrophage colony stimulating factor agonist	1

© 2022 Kammula AV et al. *JAMA Network Open.*

DNA synthesis inhibitor / radical formation stimulant	1
Thymidylate synthase inhibitor / unidentified pharmacological activity	1
B-RAF kinase inhibitor / mitogen-activated protein kinase kinase 1 inhibitor	1
ERBB-2 antagonist / immunosuppressant	1
Taxane / ERBB-2 antagonist tubulin inhibitor	1
Platelet-derived growth factor receptor kinase inhibitor / B-RAF kinase inhibitor	1
Cell cycle inhibitor / ABL receptor tyrosine kinase inhibitor	1
Angiogenesis inhibitor / epidermal growth factor antagonist	1
Vinca alkaloid / DNA inhibitor	1
DNA inhibitor / microtubule inhibitor	1
DNA synthesis inhibitor / unidentified pharmacological activity	1
Mitogen-activated protein kinase kinase 2 inhibitor / B-RAF kinase inhibitor / CTLA4 antagonist	1
RNA synthesis inhibitor / corticosteroid agonist / immunosuppressant	1
**Primary Tested Combination of Drugs: Mechanisms of Action**	**Number of Trials**
Testosterone receptor agonist / steroid synthesis inhibitor	1
17,20 lyase inhibitor / testosterone receptor agonist / androgen receptor antagonist	1
Poly ADP ribose polymerase 1 inhibitor / EGFR kinase inhibitor	1
FGF receptor 3 tyrosine kinase inhibitor / MET tyrosine kinase inhibitor / mitogen-activated protein kinase kinase 1 inhibitor	1
Taxane / ERBB-2 antagonist / iron absorption stimulant / DNA synthesis inhibitor	1
ERBB-2 antagonist / DNA inhibitor immunosuppressant	1
Poly ADP ribose polymerase 1 inhibitor / protein kinase B inhibitor	1
Ribosomal S6 kinase inhibitor / MTORC1 kinase inhibitor	1
MTORC2 kinase inhibitor / poly ADP ribose polymerase 2 inhibitor	1
CD20 antagonist / Bruton tyrosine kinase inhibitor	1
Angiogenesis inhibitor / EGFR kinase inhibitor	1
Androgen receptor antagonist / RET tyrosine kinase inhibitor	1
FGFR3 tyrosine kinase inhibitor / poly ADP ribose polymerase 2 inhibitor	1

© 2022 Kammula AV et al. *JAMA Network Open.*

DNA synthesis inhibitor / unidentified pharmacological activity / beta tubulin inhibitor	1
Taxane / granulocyte colony stimulating factor agonist / DNA inhibitor	1
Taxane / DNA inhibitor	1
RNA synthesis inhibitor / Janus kinase 1 inhibitor	1
Angiogenesis inhibitor / insulin sensitizer	1
RNA polymerase inhibitor / poly ADP ribose polymerase 2 inhibitor	1
Vinca alkaloid / BCR-Abl inhibitor	1
YES tyrosine kinase inhibitor / immunosuppressant	1
Poly ADP ribose polymerase 1 inhibitor / ATM kinase inhibitor	1
Cyclin-dependent kinase 6 inhibitor / luteinizing hormone releasing hormone (LHRH) agonist	1
Angiogenesis inhibitor / RNA synthesis inhibitor	1
Vinca alkaloid / CD20 antagonist	1
RNA synthesis inhibitor / thymidylate synthase inhibitor	1
Taxane / ERBB-2 antagonist / DNA inhibitor / DNA synthesis inhibitor	1
**Primary Tested Combination of Drugs: Mechanisms of Action**	**Number of Trials**
Taxane / ERBB-2 antagonist / DNA inhibitor / immunosuppressant	1
RNA synthesis inhibitor / luteinizing hormone releasing hormone (LHRH) agonist / estrogen receptor antagonist	1
Selective estrogen receptor modulator / ERBB-2 antagonist / poly ADP ribose polymerase 2 inhibitor	1
Mitogen-activated protein kinase kinase 2 inhibitor / mitogen-activated protein kinase kinase 1 inhibitor	1
TRKC tyrosine kinase inhibitor / ERBB-2 antagonist PD-1 antagonist	1
Protein kinase inhibitor / angiogenesis inhibitor	1
Poly ADP ribose polymerase 1 inhibitor / anaplastic lymphoma kinase inhibitor	1
Cyclin-dependent kinase 6 inhibitor / RET tyrosine kinase inhibitor	1
Hedgehog pathway inhibitor / mitogen-activated protein kinase kinase 1 inhibitor	1
Mitogen-activated protein kinase kinase 2 inhibitor / ERBB-2 tyrosine kinase inhibitor / CTLA4 antagonist	1
TRKC tyrosine kinase inhibitor / ERBB-2 antagonist	1
Immune checkpoint inhibitor / TRKA tyrosine kinase inhibitor	1

© 2022 Kammula AV et al. *JAMA Network Open.*

EGFR antagonist / EGFR kinase inhibitor	1
Apoptosis stimulant / poly ADP ribose polymerase 2 inhibitor	1
Tyrosine kinase inhibitor (TKI) / ERBB-2 antagonist / colony stimulating factor 1 receptor antagonist	1
Inosine monophosphate dehydrogenase inhibitor / DNA inhibitor / interleukin 2 antagonist	1
T cell inhibitor / DNA inhibitor	1
DNA repair enzyme inhibitor / unidentified pharmacological activity / immunosuppressant	1
CD52 antagonist / immunosuppressant	1
Angiogenesis inhibitor / CTLA4 antagonist	1
Cyclin-dependent kinase 6 inhibitor / angiogenesis inhibitor	1
Angiogenesis inhibitor / ABL receptor / Tyrosine kinase inhibitor	1
ROS receptor tyrosine kinase inhibitor / poly ADP ribose polymerase 2 inhibitor	1
Immune checkpoint inhibitor / FGF receptor 1 tyrosine kinase inhibitor	1
Immune checkpoint inhibitor / B-RAF kinase inhibitor / RET tyrosine kinase inhibitor	1
TRKC tyrosine kinase inhibitor / ERBB-2 antagonist / PI3 kinase alpha inhibitor / cyclin-dependent kinase 4 inhibitor	1
Poly ADP ribose polymerase 1 inhibitor / B-RAF kinase inhibitor / anaplastic lymphoma kinase inhibitor	1
**Primary Tested Combination of Drugs: Mechanisms of Action**	**Number of Trials**
Cyclin-dependent kinase 6 inhibitor / cyclin-dependent kinase 4 inhibitor	1
Hedgehog pathway inhibitor / anaplastic lymphoma kinase inhibitor	1
Mitogen-activated protein kinase kinase 2 inhibitor / janus kinase 1 inhibitor	1
Cell cycle inhibitor / EGFR antagonist / ERBB-2 antagonist / colony stimulating factor 1 receptor antagonist	1
VEGFR-2 tyrosine kinase inhibitor / RET tyrosine kinase inhibitor	1
MET tyrosine kinase inhibitor / anaplastic lymphoma kinase inhibitor / poly ADP ribose polymerase 2 inhibitor	1
Immune checkpoint inhibitor / ERBB-2 antagonist / PD-L1 antagonist	1
Poly ADP ribose polymerase 3 inhibitor / EGFR kinase inhibitor	1
Janus kinase 2 inhibitor / mitogen-activated protein kinase kinase 1 inhibitor	1
FGFR4 tyrosine kinase inhibitor / EGFR antagonist	1
Tyrosine kinase inhibitor (TKI) / RET tyrosine kinase inhibitor	1

© 2022 Kammula AV et al. *JAMA Network Open.*

PDGFR kinase inhibitor / poly ADP ribose polymerase 2 inhibitor	1
Mitogen-activated protein kinase kinase 2 inhibitor / VEGFR antagonist	1
Cyclin-dependent kinase 6 inhibitor / EGFR kinase inhibitor	1
Angiopoietin inhibitor / anaplastic lymphoma kinase inhibitor	1
Cell cycle inhibitor / c-KIT inhibitor	1
Angiopoietin inhibitor / cyclin-dependent kinase 4 inhibitor	1
DNA topoisomerase I inhibitor / DNA synthesis inhibitor	1
RNA synthesis inhibitor / unidentified pharmacological activity / beta tubulin inhibitor	1
Immune checkpoint inhibitor / wee-1 tyrosine kinase inhibitor / DNA inhibitor / poly ADP ribose polymerase 2 inhibitor	1
Poly ADP ribose polymerase 1 inhibitor / natural killer cell stimulant	1
Immune checkpoint inhibitor / DNA inhibitor / isocitrate dehydrogenase 2 inhibitor / cyclin-dependent kinase 4 inhibitor	1
DNA topoisomerase I inhibitor / DNA topoisomerase I inhibitor / PD-1 antagonist	1
Unidentified pharmacological activity / PD-1 antagonist	1
Taxane / granulocyte colony stimulating factor agonist / DNA inhibitor	1
Vinca alkaloid / CD20 antagonist / arachidonic acid inhibitor	1
YES tyrosine kinase inhibitor / insulin-like growth factor 1 antagonist	1
**Primary Tested Combination of Drugs: Mechanisms of Action**	**Number of Trials**
T cell inhibitor / DNA synthesis inhibitor	1
DNA repair enzyme inhibitor / histone deacetylase inhibitor	1
Apoptosis stimulant / protein synthesis inhibitor	1
Cell cycle inhibitor / janus kinase 1 inhibitor	1
Janus kinase 2 inhibitor / protein synthesis inhibitor	1
Proteasome inhibitor/ DNA synthesis inhibitor / CD3 agonist	1
Immuno-oncology therapy / LYN tyrosine kinase inhibitor	1
YES tyrosine kinase inhibitor / transcription factor NF-kappaB inhibitor	1
Proteasome inhibitor / immunosuppressant	1
RNA synthesis inhibitor / DNA synthesis inhibitor / DNA inhibitor / xanthine oxidase inhibitor / immunosuppressant	1

© 2022 Kammula AV et al. *JAMA Network Open.*

Disease modifying antirheumatic drug / CD20 antagonist / immunosuppressant / unidentified pharmacological activity	1
Immune checkpoint inhibitor / colony stimulating factor 1 receptor antagonist	1
DNA inhibitor / granulocyte macrophage colony stimulating factor agonist	1
Immunostimulant / PD-1 antagonist	1
FGFR 4 tyrosine kinase inhibitor / isocitrate dehydrogenase 1 inhibitor / anaplastic lymphoma kinase inhibitor	1
Apoptosis stimulant / mitogen-activated protein kinase kinase 1 inhibitor	1
PI3 kinase inhibitor / mitogen-activated protein kinase 1 inhibitor	1
TRKC tyrosine kinase inhibitor / enhancer of zeste homolog 2 inhibitor / MTOR kinase inhibitor	1
cyclin-dependent kinase 6 inhibitor / B-RAF kinase inhibitor	1
Mitogen-activated protein kinase 3 inhibitor / TRKA tyrosine kinase inhibitor	1
Protein kinase inhibitor / FGFR1 tyrosine kinase inhibitor	1
TRKC tyrosine kinase inhibitor / RET tyrosine kinase inhibitor	1
Immuno-oncology therapy / T cell stimulant	1
Taxane / PD-1 antagonist	1
Tyrosine kinase inhibitor (TKI) / poly ADP ribose polymerase 2 inhibitor	1
Mitogen-activated protein kinase kinase 2 inhibitor / CTLA4 antagonist	1
Cyclin-dependent kinase 6 inhibitor / anaplastic lymphoma kinase inhibitor	1
**Primary Tested Combination of Drugs: Mechanisms of Action**	**Number of Trials**
Cell cycle inhibitor / angiogenesis inhibitor	1
Apoptosis stimulant / EGFR kinase inhibitor	1
Tyrosine kinase inhibitor (TKI) ERBB-2 antagonist / colony stimulating factor 1 receptor antagonist	1
Radiopharmaceutical / poly ADP ribose polymerase 2 inhibitor	1
Wee-1 tyrosine kinase inhibitor / ataxia telangiectasia RAD3 inhibitor	1
Poly ADP ribose polymerase 1 inhibitor / ERBB-2 antagonist	1
Immune checkpoint inhibitor / polo-like kinase 4 inhibitor	1
Immune checkpoint inhibitor / wee-1 tyrosine kinase inhibitor / selective androgen receptor modulator	1
Androgen receptor antagonist / protein kinase B inhibitor MET / tyrosine kinase inhibitor / DNA inhibitor / PD-L1 antagonist	1

© 2022 Kammula AV et al. *JAMA Network Open.*

DNA inhibitor / PD-1 antagonist	1
Cell cycle inhibitor / mitogen-activated protein kinase kinase 1 inhibitor	1
ERBB-2 antagonist / unidentified pharmacological activity / DNA topoisomerase II inhibitor	1
Immune checkpoint inhibitor / PD-1 antagonist	1
DNA inhibitor / testosterone receptor agonist	1
Immune checkpoint inhibitor / testosterone receptor agonist	1
DNA inhibitor / PD-L1 antagonist	1
Proteasome inhibitor / DNA synthesis inhibitor / microtubule inhibitor	1
Vinca alkaloid / DNA topoisomerase II inhibitor / DNA synthesis inhibitor	1
Immuno-oncology therapy / DNA inhibitor	1
DNA repair enzyme inhibitor / DNA inhibitor	1
Glucocorticoid agonist / immunosuppressant CD3 agonist	1
DNA synthesis inhibitor / MDM2 inhibitor	1
Inosine monophosphate dehydrogenase inhibitor / immunosuppressant	1
DNA repair enzyme inhibitor / DNA inhibitor / immunosuppressant	1
T cell inhibitor / granulocyte colony stimulating factor agonist / immunosuppressant	1
Poly ADP ribose polymerase 1 inhibitor / steroid synthesis inhibitor	1
Radiopharmaceutical / unidentified pharmacological activity / poly ADP ribose polymerase 2 inhibitor	1
**Primary Tested Combination of Drugs: Mechanisms of Action**	**Number of Trials**
17,20 lyase inhibitor DNA inhibitor / microtubule inhibitor	1
Androgen receptor / antagonist BCL2 inhibitor	1
poly ADP ribose polymerase 1 inhibitor / bet protein inhibitor	1
EGFR kinase inhibitor / met tyrosine kinase inhibitor	1
ERBB-2 antagonist / poly ADP ribose polymerase 2 inhibitor	1
RNA synthesis inhibitor / DNA inhibitor / DNA topoisomerase I inhibitor	1
Platelet-derived growth factor receptor kinase inhibitor / ABL receptor tyrosine kinase inhibitor	1
Poly ADP ribose polymerase 1 inhibitor / MTOR kinase inhibitor	1

© 2022 Kammula AV et al. *JAMA Network Open.*

Taxane / beta tubulin inhibitor	1
Angiogenesis inhibitor / ataxia telangiectasia RAD3 inhibitor	1
Immune checkpoint inhibitor / inducible T-cell costimulator agonist	1
Cyclin-dependent kinase 6 inhibitor / insulin-like growth factor 1 antagonist	1
DNA inhibitor / vitamin b12 agonist reducing agent	1
Immune checkpoint inhibitor / ERBB-2 antagonist / unidentified pharmacological activity / CTLA4 antagonist	1
EGFR antagonist / PD-1 antagonist	1
Angiogenesis inhibitor / PD-1 antagonist	1
RNA polymerase inhibitor / PD-L1 antagonist	1
Ribonucleoside diphosphate reductase inhibitor / somatostatin receptor agonist	1
Aromatase inhibitor / poly ADP ribose polymerase 2 inhibitor	1
Interleukin 12 agonist / immunostimulant	1
RNA polymerase inhibitor / PD-1 antagonist	1
ERBB-2 tyrosine kinase inhibitor / PD-1 antagonist	1
Poly ADP ribose polymerase 1 inhibitor / ERBB-2 antagonist / EGFR kinase inhibitor	1
MET tyrosine kinase inhibitor / EGFR kinase inhibitor	1
Radiopharmaceutical / steroid synthesis inhibitor	1
17,20 lyase inhibitor / microtubule inhibitor	1
Radiopharmaceutical / testosterone receptor agonist	1
**Primary Tested Combination of Drugs: Mechanisms of Action**	**Number of Trials**
PI3 kinase alpha inhibitor / Poly ADP ribose polymerase 2 inhibitor	1
Unidentified pharmacological activity / Poly ADP ribose polymerase 2 inhibitor	1
Aromatase inhibitor / immunostimulant	1
EGFR antagonist / B-RAF kinase inhibitor	1
Angiogenesis inhibitor / PD-L1 antagonist	1
EGFR kinase inhibitor / Aurora kinase inhibitor	1

© 2022 Kammula AV et al. *JAMA Network Open.*

eFigure 1. End Points in Oncology Trials Using Germline Data for Eligibility vs. Trials Not Using Germline Data

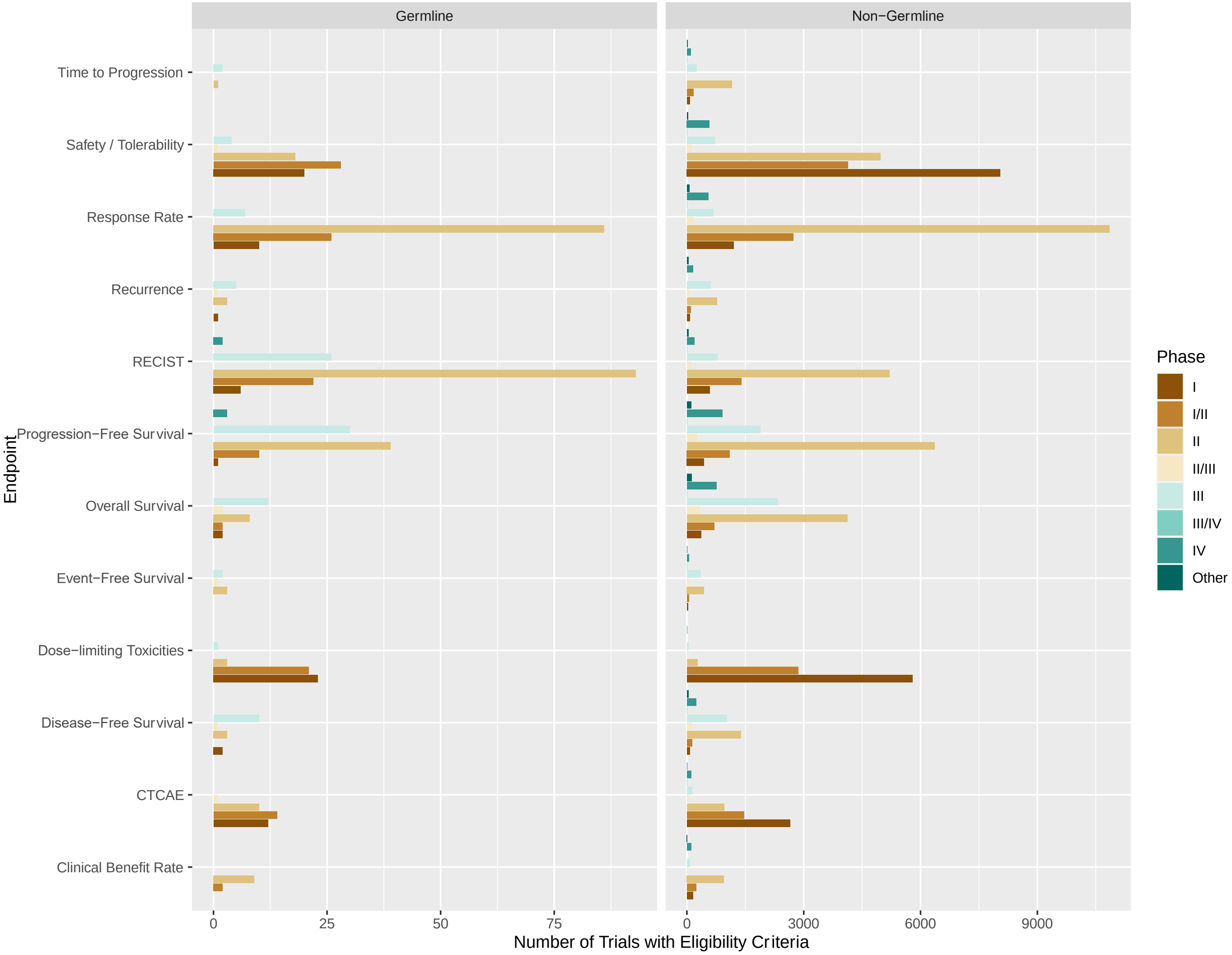

Distribution of clinical trials in Trialtrove by end points and phases. Left graph in figure shows the distribution of accrual in oncology trials using germline data for eligibility criteria, while right graph in figure shows the distribution of accrual in oncology trials that do not use germline data at all. Number of trials is shown on the X-axis, and endpoint types are shown on the Y-axis.

© 2022 Kammula AV et al. *JAMA Network Open.*

eFigure 2. Outcomes in Oncology Trials Using Germline Data for Eligibility vs. Trials Not Using Germline Data

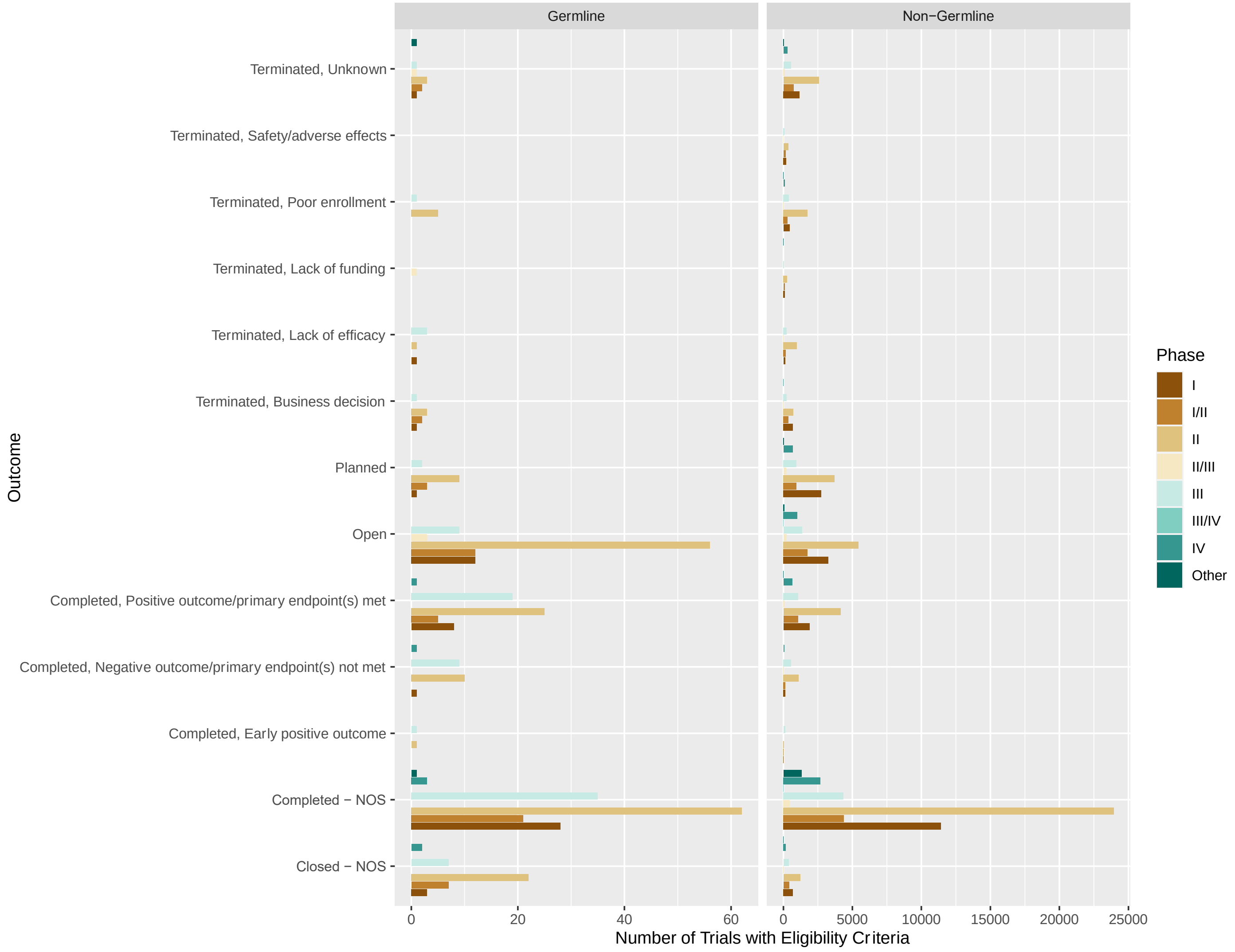

Distribution of clinical trials in Trialtrove by outcomes and phases. Left graph in figure shows the distribution of accrual in oncology trials using germline data for eligibility criteria, while right graph in figure shows the distribution of accrual in oncology trials that do not use germline data at all. Number of trials is shown on the X-axis, and endpoint types are shown on the Y-axis.

© 2022 Kammula AV et al. *JAMA Network Open.*

eFigure 3. Percent Accrual in Oncology Trials Using Germline Data for Eligibility vs. Trials Not Using Germline Data

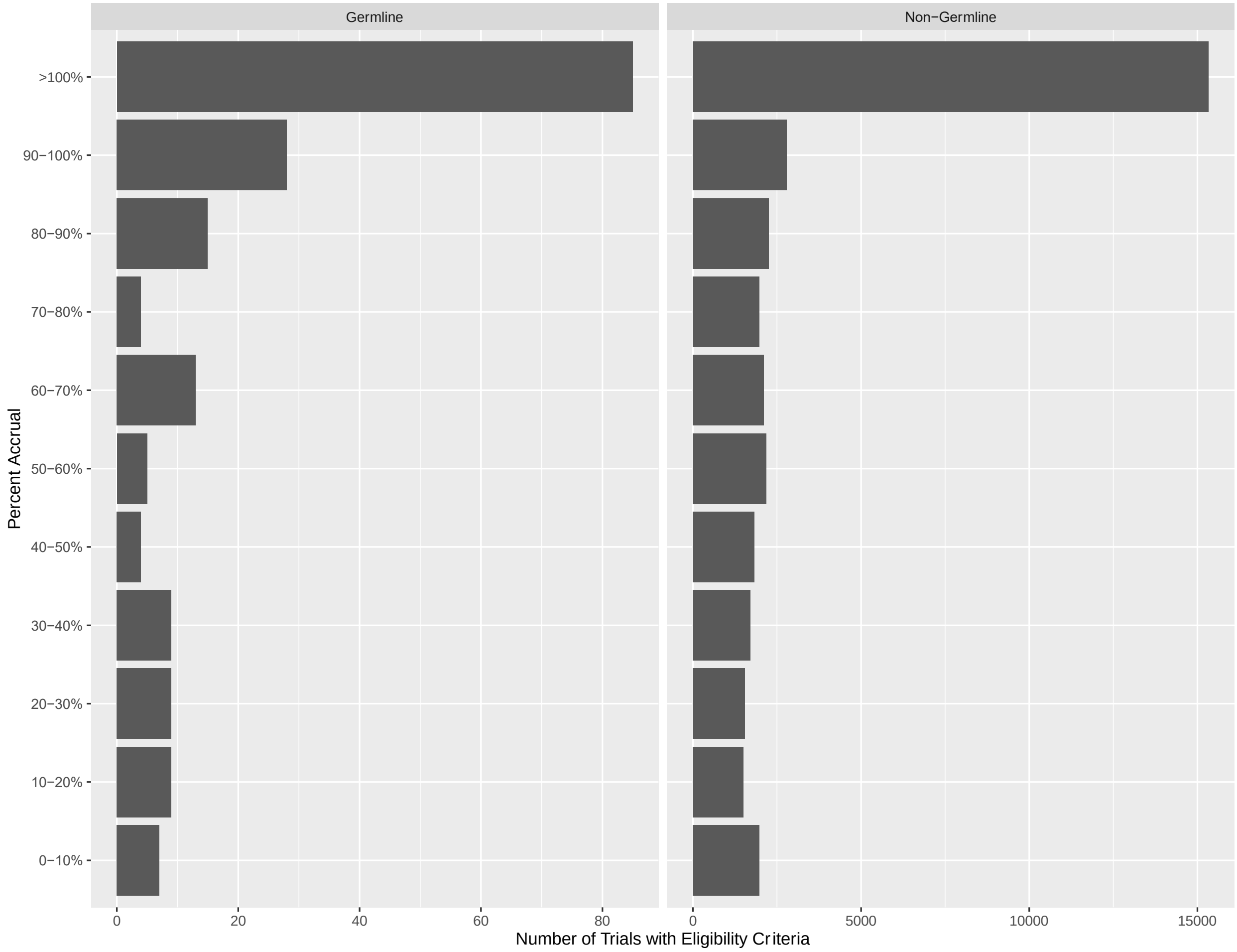

Distribution of percent accrual of clinical trials in Trialtrove. Left graph in figure shows the distribution of accrual in oncology trials using germline data for eligibility criteria, while right graph in figure shows the distribution of accrual in oncology trials that do not use germline data at all. Number of trials is shown on the X-axis, and percent accrual is shown as a categorical variable on the Y-axis.

© 2022 Kammula AV et al. *JAMA Network Open.*
